# A Case Study to Dissect Immunity to SARS-CoV-2 in a Neonate Nonhuman Primate Model

**DOI:** 10.3389/fimmu.2022.855230

**Published:** 2022-05-04

**Authors:** Claire-Maëlle Fovet, Camille Pimienta, Mathilde Galhaut, Francis Relouzat, Natalia Nunez, Mariangela Cavarelli, Quentin Sconosciuti, Nina Dhooge, Ilaria Marzinotto, Vito Lampasona, Monica Tolazzi, Gabriella Scarlatti, Raphaël Ho Tsong Fang, Thibaut Naninck, Nathalie Dereuddre-Bosquet, Jérôme Van Wassenhove, Anne-Sophie Gallouët, Pauline Maisonnasse, Roger Le Grand, Elisabeth Menu, Nabila Seddiki

**Affiliations:** ^1^Université Paris-Saclay, INSERM, CEA, Center for Immunology of Viral, Auto-immune, Hematological and Bacterial Diseases (IMVA-HB/IDMIT), Fontenay-aux-Roses, France; ^2^Life and Soft, Fontenay-aux-Roses, France; ^3^Diabetes Research Institute, IRCCS Ospedale San Raffaele, Milan, Italy; ^4^Viral Evolution and Transmission Unit, Division of Immunology, Transplantation and Infectious Diseases, IRCCS Ospedale San Raffaele, Milan, Italy; ^5^MISTIC Group, Department of Virology, Institut Pasteur, Paris, France

**Keywords:** SARS-CoV-2, innate immunity, type-I IFN, pediatric, neonate, children, COVID-19, nonhuman primate, microbiota

## Abstract

Most children are less severely affected by coronavirus-induced disease 2019 (COVID-19) than adults, and thus more difficult to study progressively. Here, we provide a neonatal nonhuman primate (NHP) deep analysis of early immune responses to severe acute respiratory syndrome coronavirus 2 (SARS-CoV-2) infection in blood and mucosal tissues. In addition, we provide a comparison with SARS-CoV-2-infected adult NHP. Infection of the neonate resulted in a mild disease compared with adult NHPs that develop, in most cases, moderate lung lesions. In concomitance with the viral RNA load increase, we observed the development of an early innate response in the blood, as demonstrated by RNA sequencing, flow cytometry, and cytokine longitudinal data analyses. This response included the presence of an antiviral type-I IFN gene signature, a persistent and lasting NKT cell population, a balanced peripheral and mucosal IFN-γ/IL-10 cytokine response, and an increase in B cells that was accompanied with anti-SARS-CoV-2 antibody response. Viral kinetics and immune responses coincided with changes in the microbiota profile composition in the pharyngeal and rectal mucosae. In the mother, viral RNA loads were close to the quantification limit, despite the very close contact with SARS-CoV-2-exposed neonate. This pilot study demonstrates that neonatal NHPs are a relevant model for pediatric SARS-CoV-2 infection, permitting insights into the early steps of anti-SARS-CoV-2 immune responses in infants.

## Introduction

Severe acute respiratory syndrome coronavirus 2 (SARS-CoV-2)-infected children are often asymptomatic or develop mild symptoms, and thus, compared with adults, are less frequently in need of hospitalization and show a lower mortality rate (1–5). Dysregulated innate immune responses, such as anti-interferon (IFN) antibodies or delayed responsiveness, have been reported in some severe COVID-19 cases but cannot account for the majority of severe infections (6–8). A rare and serious postinfectious condition that can occur 2–6 weeks after SARS-CoV-2 infection, termed pediatric inflammatory multisystem syndrome (PIMS-TS) or multisystem inflammatory syndrome (MIS-C), has been reported (9). Several hypotheses have been proposed to explain why children are protected from more severe outcomes with COVID-19 compared with adults, although not always conclusive. These include differences in the expression of the angiotensin-converting enzyme 2 (ACE2) receptor resulting in lower viral RNA loads (10–12), the presence of antibodies to common cold coronaviruses that might provide partial protection (6, 7), and a robust innate response early in the course of infection (8, 13–16). This latter explanation seems more robust and would clarify why children are less affected by COVID-19 (12), but further investigations are needed to fully support this.

Longitudinal data from early viral and immunological events following SARS-CoV-2 infection of children are difficult to obtain, and the reason is often linked to symptoms onset that go unnoticed in this population (1–4). Moreover, concomitant sample collection from the blood, lungs, and gastrointestinal compartments where the virus can be found, is difficult to perform in humans. Thus, the use of a preclinical pediatric model is valuable in this context. Several animal species were evaluated as models of initial SARS-CoV and MERS-CoV human diseases and while most laboratory animals, including mice, hamsters, ferrets, and nonhuman primates (NHP), could be productively infected, only a few species displayed overt clinical disease without requirement of adaptation of viral strains to the animals (17). Macaques have been shown to be reproducibly susceptible to infection to coronaviruses affecting humans and develop acute respiratory syndrome recapitulating the disease (18–26). Because of their phylogenetic proximity, macaques share a similar organization of the immune system with humans. Regarding SARS-CoV-2, ACE2 is expressed in both humans and macaques, with similar distribution and functionalities (13, 19). In recent studies, we have shown that adult cynomolgus macaques reliably develop infection upon intranasal and intratracheal exposure, and mild to moderate lesions were observed in the lungs during the first-week postchallenge, similar to human cases (27, 28).

Here, we describe an experimental SARS-CoV-2 infection in neonate NHP in which we longitudinally studied for 2 months viral kinetics, innate and adaptive immune responses, and microbiota profiles in different compartments, including blood, nasal, oropharyngeal, gastrointestinal, ocular, and vaginal sites. The neonate developed an asymptomatic infection, while its unexposed breastfeeding mother exhibited a low viral RNA load close to the quantification limit, despite their very close contact. Peak viral load in the neonate correlates with the development of an early innate immune response with an IFN gene signature, as demonstrated by RNA sequencing, flow cytometry, and cytokine longitudinal data analyses. Viral kinetics and immune responses correlate with changes in the microbiota profile composition in the oropharyngeal and rectal mucosae. Altogether, these findings support the use of neonate NHP as a suitable model to get insights into early pathogenic mechanisms of the human pediatric SARS-CoV-2 infection.

## Materials and Methods

### Animals

A healthy pregnant female rhesus macaque (*Macaca mulatta*), 6 years old with a body weight of 6 kg, was imported from the *Station de Primatologie* (CNRS, Rousset-sur-Arc, France). Pregnancy was monitored until vaginal delivery of a full-term healthy female neonate. No complications were noted during delivery or subsequent breastfeeding. Seven adult female rhesus macaques (Hartelust, Tilburg, Netherlands), from three different studies conducted in our laboratory, aged 4–5 years old with a body weight of 5–7 kg, were also used in this study.

All animals were housed in the BSL3 facilities of the Infectious Disease Models and Innovative Therapies (IDMIT) infrastructure (CEA, Fontenay-aux-Roses, France). CEA complies with French national regulation (facilities authorization number #D92-032-02), the European Directive 2010/63/EU, and the Standards for Human Care and Use of Laboratory Animals (OLAW animal welfare assurance number #A5826-01, United States). The study was approved by the local ethical committee (CEtEA#44) and the French Research, Innovation and Education Ministry under registration number APAFIS #24434-20200030216532863 V3.

For all procedures, the mother was sedated with ketamine (Imalgene 1000, 10 mg/kg, Rhône-Mérieux, France)/medetomidine (Domitor, 0.5 mg/kg, Vetoquinol, France) to access the neonate. The neonate was either sedated for imaging or blood collection or restrained for fluid collection, the neonate was anesthetized with ketamine (5 mg/kg) and medetomidine (0.5 mg/kg). Anesthesia was reversed with atipamezole 2.5 mg/kg (Antisedan^®^, Vetoquinol, France) first in the neonate until fully awaken, then in the mother. Both were monitored until complete recovery and total reattachment. Adult females were anesthetized following the same procedure. The volume of blood sampling per week for both the neonate and mother was performed according to ethics and protocols.

### Study Design and Clinical Follow-Up

**Figure 1:**
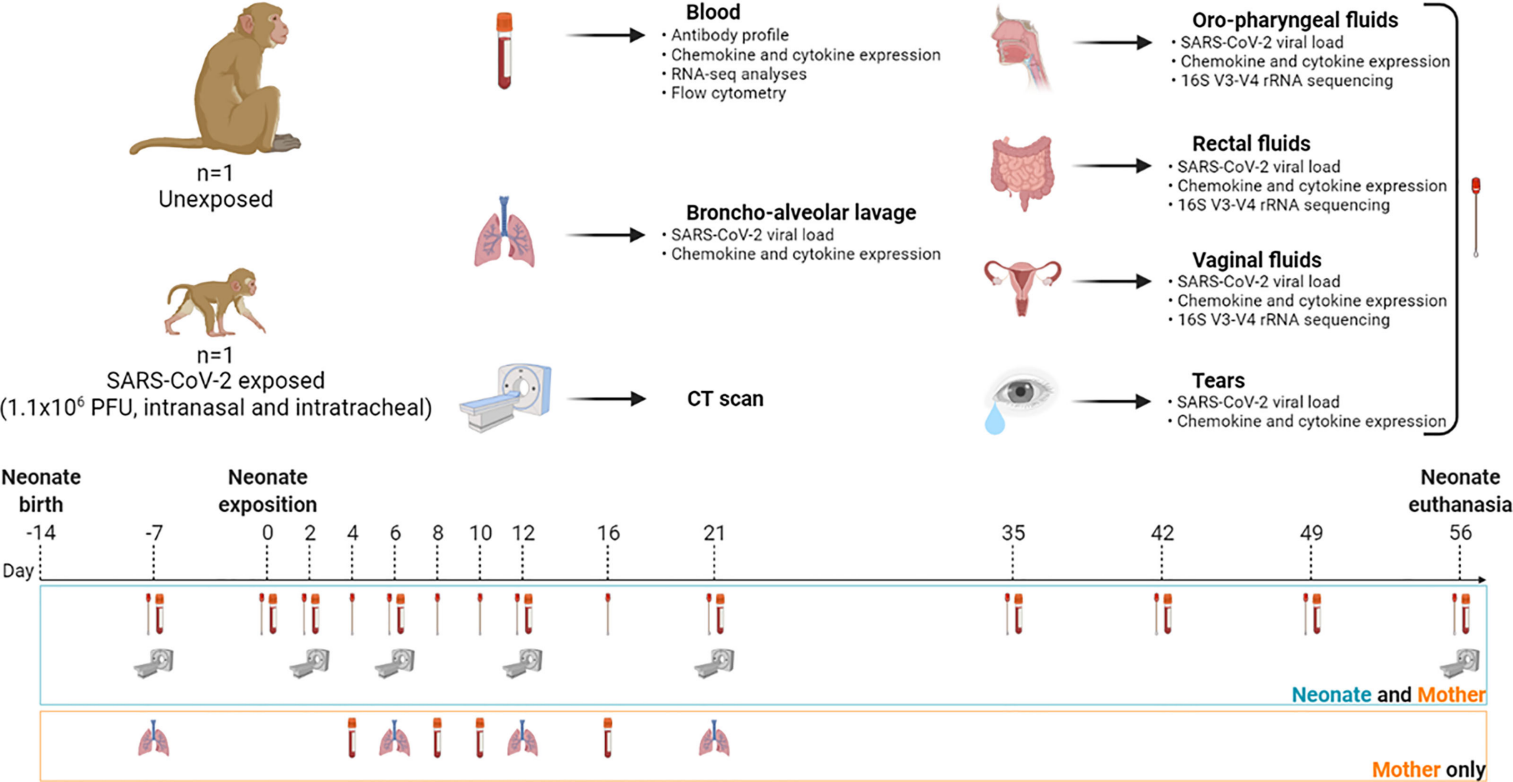
Study design and workflow. A female rhesus macaque neonate was exposed at 14 days of life to a total dose of 1.1 × 10^6^ PFU of SARS-CoV-2 by the intranasal and intratracheal routes. Symbols: blood tube for bleeding; lungs for bronchoalveolar lavage; scan machine for CT session; swab for biological fluid collection (nasal, oropharyngeal, vaginal, rectal, and tears). Samples were collected at day postinfection (DPI) −7, 0, 2, 4, 6, 8, 10, 12, 16, 21, 35, 42, 49, and 56. Analysis of peripheral blood and fluids included viral RNA load kinetics and chemokine/cytokine quantification. Microbiota identification was done in every fluid compartment. Antibody profiling, RNA-seq analyses, and flow cytometry were also performed in blood. Created with BioRender.com.

The longitudinal follow-ups are summarized in **Figure 1** and **Supplementary Table S1** for the neonate/mother pair and adult females, respectively. Animals were observed 7 days a week, and any abnormal behavior was reported in a specific individual file. Clinical examination, body weight, rectal temperature, oxygen saturation, and respiratory and heart rates were recorded at each bleeding. Blood and biological fluids (nasal, vaginal, rectal, oropharyngeal, and tears) were collected for the neonate/mother pair at day postinfection (DPI) −7, 0, 2, 4, 6, 8, 10, 12, 16, 21, 35, 42, 49, and 56. To limit collected blood volume and frequency of sedation, the neonate was not anesthetized nor bled at DPI 4, 8, 10, and 16. Only fluids were sampled under restraint at these time points. Bronchoalveolar lavages were performed on the mother at DPI −7, 6, 12, and 21. Blood and biological fluids (nasal, rectal, bronchoalveolar lavages) were collected from adult females according to the schedule of their respective studies and described in **Supplementary Table S1**. Complete blood count was determined using an HMX A/L analyzer (Beckman Coulter, USA) and biochemical parameters (C-reactive protein, haptoglobin, creatinine, urea, alanine–aspartate–aminotransferase (ASAT/ALAT), lactate dehydrogenase (LDH), troponin I, and total proteins) were assessed using an ADVIA1800 analyzer (Siemens). After 2 months, the neonate was euthanized with an intravenous administration of 180 mg/kg of sodium pentobarbital (Dolethal, Vetoquinol, France) under anesthesia.

### SARS-CoV-2 Virus and Infection

SARS-CoV-2 virus (hCoV-19/France/lDF0372/2020 strain, passaged twice in Vero E6 cells) was provided by the National Reference Centre for Respiratory Viruses (Institut Pasteur) as previously described (23). Neonate macaque was inoculated 14 days after birth with 1.1 × 10^6^ plaque-forming units (PFU) of SARS-CoV-2 and adult females with 1 × 10^5^, 1 × 10^6^, or 1 × 10^7^ (see **Supplementary Table S1**) through a combination of intranasal and intratracheal routes after a premedication of atropine sulfate 0.04 mg kg^−1^ (Aguettant, France) and under anesthesia. The challenge took place under a class II biological safety cabinet with the animal placed in a supine position.

Virus preparation was applied slowly into each nostril (50 or 250 µl each for neonates and adults, respectively) using a 100-µl micropipette. The nostrils were slowly massaged for 1 min. After nasal exposure using a laryngoscope to visualize the epiglottis and larynx, a 1-mm diameter bladder catheter (neonate, ref#706537, Coveto, France) or a 3.5-mm diameter endotracheal probe (adults, ref#647587, Coveto, France) was introduced and stopped before the carina, then connected to a 1-ml syringe to apply the virus into the trachea (450 µl or 4.5 ml for neonate and adults, respectively). A 1-ml syringe of air was injected inside the probe to make sure that all virus preparation was injected. The animal’s face was wiped to clean off any residual virus. Oxygen saturation and heart rate were monitored for 10 min after inoculation. Anesthesia was reversed first in the neonate, then in the mother, as described above.

### Chest CT *In Vivo* Imaging

Chest computed tomography (CT) scans were performed on neonate and mother at DPI −7, 2, 6, 12, 21, and 56, as described in **Figure 1**. All imaging acquisitions were performed on a CT system (Vereos-Ingenuity, Philips) as previously described (27–30). CT scans were performed using the following parameters: CT detector collimation of 64 × 0.6 mm, tube voltage of 80 kV (neonate) or 120 kV (mother and adult animals), and intensity of about 350 mAs (neonate) or 150 mAs (mother and adult animals), 1.25-mm slice sickness, and 0.25-pixel spacing. Animals were placed in a supine position with thermal support (Bear Hugger, 3M) on the machine bed with heart rate, oxygen saturation, and temperature monitoring. Pulmonary lesions were defined as ground-glass opacity, crazy-paving pattern, or consolidation as previously described (29, 30). Lesion features detected by CT imaging were blindly assessed by two persons independently, and final CT score results were reached by consensus.

### Viral Quantification

Viral RNA loads were assessed for both mother and neonate in nasal, oropharyngeal, vaginal, and rectal fluids, tears, and maternal bronchoalveolar lavages by RT-qPCR with a plasmid standard concentration range containing an RdRp gene fragment, including the RdRp-IP4 RT-PCR target sequence. Viral RNA loads in adult females were determined in nasal, rectal, and bronchoalveolar lavage fluids when available (see **Supplementary Table S1**), following the same procedure. The protocol describing the procedure for the detection of SARS-CoV-2 is available on the WHO website (https://www.who.int/docs/default-source/coronaviruse/real-time-rt-pcr-assays-for-the-detection-of-sars-cov-2-institut-pasteur-paris.pdf?sfvrsn=3662fcb6_2). The limit of detection was estimated at 2.37 log10 copies/ml, and the limit of quantification was estimated at 3.37 log10 copies/ml.

#### Lentiviral Vector-Based SARS-CoV-2 Neutralization Assay

Plasmid pSpike-C3 expressing the codon-optimized SARS-CoV-2 Spike protein open reading frame (ORF) (GenBank: NC_045512.2) containing a 21-amino acid deletion at the cytoplasmic tail (delta21) of Spike protein was previously described (31). The Lenti-X 293T human embryonic kidney cell line (Clontech, Mountain View, CA, USA) was used for the production of LV-Luc pseudotyped with Wuhan-Hu-1 Spike by transient transfection. LV-Luc preparations were titered on VeroE6 cells (African green monkeys, epithelial kidney), and dilutions providing 150,000–200,000 relative luciferase units (RLU) were used in the neutralization assay. Briefly, heat-inactivated serum serial 3-fold dilutions starting from the 1/40 dilution for the adult macaque and 1/20 for the neonate were incubated in duplicate with the LV-Luc for 30 min at 37°C in 96-well plates and thereafter added to VeroE6 cells at a density of 20,000 cells/well. After 48 h, luciferase expression was determined with a luciferase assay system (Bright-Glo, Promega) and measured in a Mithras luminometer (Berthold, Germany). The 50% inhibitory serum dilution (ID50) was calculated with a linear interpolation method using the mean of the duplicates (32). Neutralization was expressed as the reciprocal of the serum dilution giving 50% inhibition of RLU compared with the mean of the virus control wells. An ID50 below the lowest serum dilution used was considered negative.

#### IgG-Binding Antibody Luciferase Immunoprecipitation System Assay

Using luciferase immunoprecipitation system (LIPS) (33), we measured IgG binding to recombinant nanoluciferase-tagged antigens corresponding to SARS-CoV-2 Wuhan-Hu-1 spike RBD domains, as previously described (34). Viral sequences used in this study corresponded to the deposited sequence Genebank NC_045512.2 for SARS-CoV-2 Wuhan. Briefly, we cloned recombinant nanoluciferase-tagged antigens and expressed them by transient transfection into Expi293F™ cells (Expi293™ Expression System, ThermoFisher Scientific Life Technologies, Carlsbad, CA, USA). For LIPS, we incubated in liquid phase each antigen with test serum (1 µl) for 2 h and then captured immune complexes with rProtein A-sepharose. After washing (5 times) the sepharose pellets, we quantified bound IgG by measuring the recovered luciferase activity in a Berthold Centro XS3 luminometer (Berthold Technologies GmbH & Co. KG, Bad Wildbad, Germany) using the MikroWin version 5.22 software. We then converted raw data into arbitrary units (AU), using a local positive index serum for SARS-CoV-2-specific antibodies.

### Flow Cytometry

Immune profiling analysis was performed on fresh heparin–lithium whole blood by flow cytometry. Two antibody panels (myeloid/lymphoid) were established, using for each one 20 or 50 µl of blood from the neonates and the adults, respectively. Staining was performed with Blue-Vid for cell viability (Invitrogen) and anti-human antibodies that cross-react with NHP antigens, including CD45, CD3, CD4, CD95, CD69, HLA-DR (Becton Dickinson), CD8 (Miltenyi, Germany), and CD20 (Fisher Scientific). After two washes, erythrocytes were lysed and PBMCs were fixed (paraformaldehyde (PFA)) before acquisition with an LSRII flow cytometer (BD Bioscience, USA). Analyses were performed using FlowJo software (version 10.7.1; Treestar Inc., CA, USA).

### Cytokines Analyses

Cytokine quantification (25 μl/sample) was performed for neonate and its mother in serum and fluids (nasal, oropharyngeal, rectal, and vaginal) and in the serum of adult females with the Milliplex MAP kit for nonhuman primates, which is based on Luminex^®^ xMAP multiplex technology for the detection of 23 cytokines: G-CSF, GM-CSF, IFNγ, IL-1β, IL-1Ra, IL-2, IL-4, IL-5, IL-6, IL-8, IL-10, IL-12/23, IL-13, IL-15, IL-17A, MCP-1, MIP-1α, MIP-1β, sCD40L, TGF-α, VEGF, and IL-18, according to manufacturer’s instructions.

### RNA Sequencing

Frozen whole blood Tempus samples were processed for RNA extraction using Tempus Spin RNA Isolation Kit (Applied Biosystems, USA). RNA was then concentrated using RNA Clean XP beads (Beckman Coulter, USA). RNA was quantified using QuBit (ThermoFisher), and a quality check was performed on the Agilent TapeStation system. A total of 100 ng of RNA per sample was denatured at 65°C and retrotranscribed by a strand-switching technique using Maxima H Minus Reverse Transcriptase (ThermoFisher, USA) to synthesize a double-stranded cDNA. PCR, barcode, and adapter attachment were performed according to SQL-PCB109 cDNA-PCR Sequencing Kit (Oxford Nanopore Technologies, Oxford, UK). Samples were quantified using QuBit dsDNA HS (ThermoFisher, USA) kit before loading on R9.4.1 Flow cells using the GridION instrument.

### Transcriptome Analysis

Sequence reads were converted into FASTQ files. Reads under 100 bp or with a quality score under seven were discarded. The remaining reads were aligned on the *Macaca mulatta* transcriptome of reference (GeneBank assembly accession number GCA_003339765.3) using minimap2 (35) version 2.17. To quantify transcripts, the resulting alignments were given to Salmon version 1.4.0 (36). To explore single replicates, samples were duplicated to use DESeq2 version 1.32.0 (37).

We performed a gene set enrichment analysis with both upregulated and downregulated genes Log2FC>2 or Log2FC<2, respectively, using Enrichr, a web server enrichment analysis tool (38–40), and BioPlanet 2019 database for cellular and signaling pathway analysis (41). To determine the type-I IFN protein-protein association network and identify a type-I IFN signature for innate response at DPI 2, we used the STRING database, a web resource of known and predicted gene-gene or protein-protein interactions (https://string-db.org/). The network predicts associations for a particular gene dataset (42). More lines between the nodes, more types of evidence found in the interaction.

Of note, since differential expression with DESeq2 version 1.32.0 (37) requires at least two biological replicates for each condition, which was not possible for this part of the study, the samples were artificially duplicated for further analysis. The differentially expressed genes were thus, considered exploratory. Nevertheless, the several longitudinal time points available enabled us to analyze and interpret the data by comparison with the literature.

### Microbiota Sequencing

DNA from the oropharyngeal, rectal, and vaginal swabs were extracted using the PowerFecal DNA Pro Kit (Qiagen^®^, Germany) following the manufacturers’ instructions. Purified DNA was quantified using a QuBit fluorometer. Samples were concentrated with the magnetic AMPure XP beads (Beckman Coulter) and stored at −20°C until use.

Sequencing of the V3–V4 region of the 16S rRNA gene was performed using the 16S Metagenomic Sequencing Library Preparation protocol for MiSeq System (Illumina). Briefly, 16S rRNA V3–V4 regions were amplified using 5′ CCTACGGGNGGCWGCAG and 5′ GACTACHVGGGTATCTAATCC primers with overhanging adapters. Amplicons were purified to remove free or dimerized primers with the AMPure XP beads (Beckman Coulter). Dual Indexes and Illumina sequencing adapters were attached using TrueSeq Index Plate (Illumina). Samples were quantified using QuBit, and a 4nM library was denatured. PhiX library was integrated as an internal control. The library was sequenced using the MiSeq device (Illumina).

### Metagenomics Sequencing Data Processing and Taxonomic Assignation

FASTQ sequences were processed using the Find Rapidly OTU with Galaxy Solution (FROGS) pipeline (43) implemented on a galaxy instance (http://migale.jouy.inra.fr/galaxy/). Bacterial 16S rRNA-matched readings were merged with a maximum rate of 0.1 mismatches in the overlapping region using Vsearch (44). Each of the samples was a unique time point; thus, after dereplication, the clusterization step ran with an aggregation distance equal to 1 (maximum number of differences between all of our sequences), and thus denoising was not needed. Chimeras were removed using Vsearch, and 99% of the total sequence abundance was kept. OTUs with less than 0.00001% abundance were filtered out. Finally, taxonomic affiliation was performed using SILVA 138 pintail 100 databases. Data normalization was performed to the lowest sequencing depth using both mother and neonate reads.

### Statistical Analysis

We used GraphPad Prism 8.0.2 software to analyze viral and immunological data as well as microbiota OTUs and bacterial abundance and cytokine quantification. The mean value of normalized read counts was calculated for each taxon. Taxa having less than 1% of relative mean abundance were assigned to the category “Others”. All relative mean abundances are represented in cumulative histograms. For correlations between cytokines, viral RNA load, and microbiota, a nonparametric test of Spearman was performed, heatmaps were generated based on the obtained R correlation factors, and one or more (^*^) are shown according to the *p*-value indicated in the figure legends. Percentages of cell populations from flow cytometry analyses and cytokines were expressed as a fold change from the baseline at DPI −7 for each time point. The log of fold change was indicated in the figures when applied.

## Results

### Clinical Parameters

After infection, the neonate exhibited asymptomatic clinical signs and had no major changes in temperature, oxygen saturation, respiratory rate, and heart rate (**Supplementary Figure S1A**). The newborn weighed around 450 g at birth and gained weight throughout the study, consistent with expected standards (45) (**Supplementary Figure S1B**).

As previously reported (46), we observed elevated levels of creatinine, ASAT, and LDH at baseline, followed by a physiological decrease as early as the second week of life. Interestingly, we detected an increase in the ASAT and LDH levels at DPI 2 in the neonate (**Supplementary Figure S1C**).

No variations in the different leukocyte subpopulations, red blood cells, and hemoglobin were observed (**Supplementary Figure S2A**). The mother showed lymphocytosis and neutropenia at DPI 2, probably due to the stress of the manipulations.

Overall, this follow-up of clinical monitoring showed normal myeloid and lymphoid cell population counts at different time points in the neonate and the mother. As expected, we observed an inverse lymphocyte to neutrophil count ratio between neonate and adult blood that started to normalize at 10 weeks of life (47) (**Supplementary Figure S2B**).

As for the infected adult females, we did not see any symptoms nor variation in the clinical parameters (**Supplementary Figure S3A**). However, we did observe signs of inflammation through increased levels of hepatic transaminases and C-reactive protein (CRP) throughout their follow-up, and a transient lymphopenia at DPI 2 (**Supplementary Figures S3B****,** **S4**, respectively).

### SARS-CoV-2 Neonate Infection and Follow-Up

**Figure 2:**
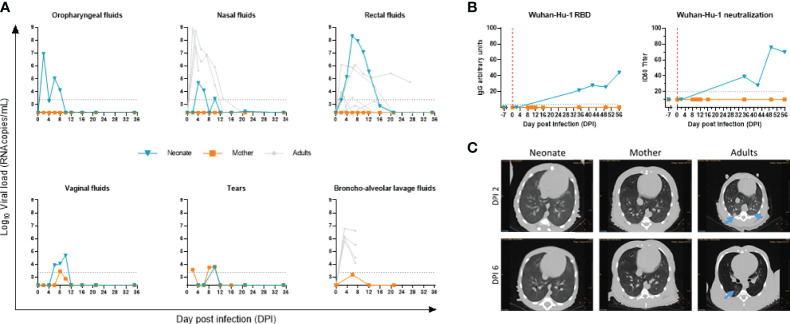
Follow-up of SARS-CoV-2 infection. **(A)** Viral RNA load measured by RT-qPCR in the oropharyngeal, nasal, rectal, vaginal, tears, and bronchoalveolar fluidic compartments. The blue line is for the neonate, orange line for the mother, and gray lines for adult NHPs. The limit of detection was estimated at 2.37 log10 copies/ml, and the limit of quantification was estimated at 3.37 log10 copies/ml (dotted horizontal line). **(B)** Line plots of RBD IgG arbitrary units (AU) and 50% inhibitory serum dilution (ID50) against SARS-CoV-2 Wuhan-Hu-1 in sequential serum samples of the neonate. **(C)** Thoracic CT scan at DPI 2 (upper panel) and DPI 6 (lower panel) for neonate (left), mother (middle), and infected adult (right). Blue arrows point to ground-glass opacities.

The neonate had a high viral RNA load, i.e., 7 × log10 copies/ml as early as DPI 2 in the oropharyngeal samples, as evaluated by RT-qPCR genomic viral RNA. The viral RNA load progressively decreased to undetectable levels from DPI 10 and remained undetectable until the end of the follow-up. The virus was also detected in the neonate’s rectal fluids with higher levels than those of adults (**Figure 2A**). In nasal fluids, the viral RNA load peaked at almost 5 × log10 copies/ml. Adult animals, displaying no differences between virus doses tested (**Supplementary Figure S5**), showed similar viral RNA load kinetics in nasal fluids, although at a higher level (**Figure 2A**). The anatomical differences between adults and newborns might explain the variation in viral RNA load detection, given that we barely entered the nostril in the newborn while we reached the nasal turbinates in adults. Finally, in vaginal fluids and the tears, the viral RNA load was lower than in oropharyngeal and rectal fluids, peaking at almost 5 and 4 × log10 copies/ml, respectively (**Figure 2A**).

To exclude a potential transmission of infection from the neonate to the mother, we tested her virus load. Only tears and vaginal fluids showed a minimal positive signal above the detection limit, while no virus was detected above the detection limit in the oropharyngeal, nasal, rectal, or bronchoalveolar space compartments (**Figure 2A**).

Overall, these data indicate that the newborn was infected in the respiratory tract. As for the potential infection of the digestive tract, we observed a high rectal viral RNA load that persisted over time with a slower clearance compared with adults. Whether it is the result of the swallowing of viral particles that replicated in the throat or an active infection of SARS-CoV-2 in the gastrointestinal tract needs to be confirmed. As for the mother, despite the close contact with the baby, especially during breastfeeding, we did not detect the presence of SARS-CoV-2 by RT-qPCR above the detection limit in the respiratory or intestinal tracts (**Figure 2A**).

We next assessed the kinetics of antibodies to SARS-CoV-2. We used the lentiviral vector-based SARS-CoV-2 neutralization assay and the LIPS assays to profile the antibody response to spike antigen of SARS-CoV-2 Wuhan-Hu-1. IgG to RBD and neutralizing antibody to SARS-CoV-2 were negative at baseline DPI −7 and DPI 2 in the neonate, while the sera from DPI 35 onwards showed a gradual increase (**Figure 2B**). In agreement with the low to undetectable viral RNA load assessed by RT-qPCR, no antibody response was detected in the maternal serum samples.

To characterize lung lesions, we used *in vivo* imaging at crucial time points postinfection. No pulmonary changes were observed in the neonate’s lungs, as evidenced by the CT scan (**Figure 2C**, left image), compared with what can be found in the adult’s lungs (right images) at DPI 2 and DPI 6. SARS-CoV-2-infected adult animals that developed a mild infection after virus inoculation showed ground-glass opacities by CT scan (**Figure 2C**, right panel and blue arrows), especially in the dorsal area of the middle and lower lobes. No lesions were observed in the mother.

**Figure 3:**
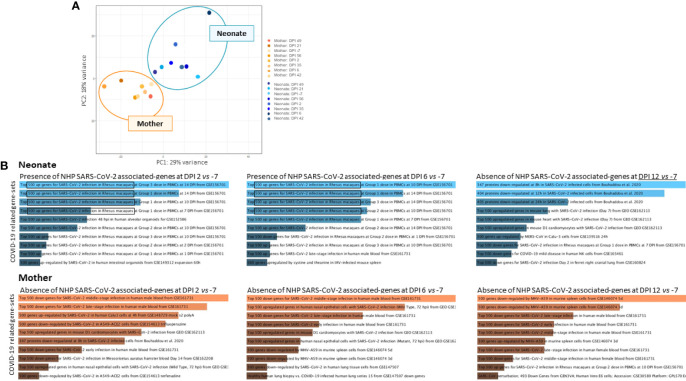
RNA-sequencing analyses of whole blood. RNA-sequencing experiments on longitudinal whole blood samples (DPI −7, 2, 6, 12, 21, 35, 42, 49, and 56) to detect the presence of COVID-19-associated gene sets. **(A)** Principal component analysis (PCA) of neonate samples (blue) and mother samples (orange) show no association between individuals where mother and neonate samples are presented. **(B)** Increased host-responding genes to SARS-CoV-2 infection at DPI 2 and DPI 6 compared with the baseline at DPI −7 were observed, in the neonate (upper panel in blue) but not in the mother (lower panel in orange). SARS-CoV-2-associated genes decrease at DPI 12 in the neonate.

In addition to RT-qPCR and CT scans that showed the presence of virus and signs of infection, respectively, we sought to use RNA-sequencing data analysis to reveal the presence of host responding genes to COVID-19 in longitudinal whole blood samples in the neonate compared with the mother. First, our analysis indicated no association and a sharp distinction between neonate and mothers’ gene sets, as demonstrated by the principal component analysis (PCA) (**Figure 3A**). Further data analyses showed an increase in host responding genes to SARS-CoV-2 infection at DPI 2 and DPI 6 compared with the baseline in the neonate (**Figure 3B**, upper panel) but not in the mother (**Figure 3B**, lower panel), adding a further level of evidence for infection.

### SARS-CoV-2-Exposed Neonate Shows an Early Type-I IFN Response in the Blood

Using an RNA-sequencing-based approach, we aimed to reveal the very early antiviral gene signature that emerges following SARS-CoV-2 exposure using longitudinal neonate’s whole blood.

**Figure 4:**
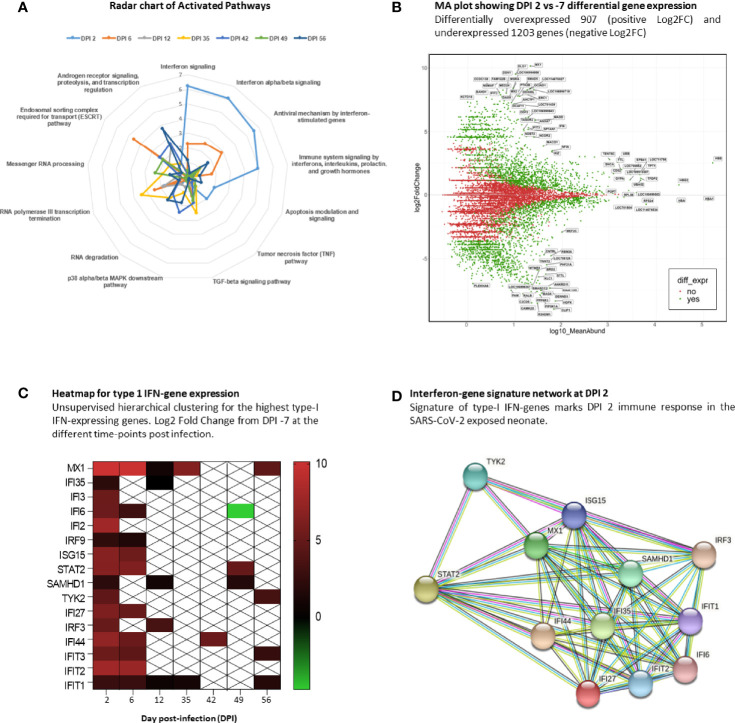
Interferon signature at DPI 2. RNA-sequencing analysis of neonate whole blood. **(A)** Radar chart of upregulated genes at the infected time points and their associated activation pathways. **(B)** MA plot of DPI 2 showing 2,110 differentially expressed genes in green, with 907 upregulated and 1,203 downregulated genes. **(C)** Heatmap showing the gene-expression level of type-I IFN signature genes at different time points. White crossed rectangles are for no gene-expression changes between corresponding time points. **(D)** The STRING network inference for type-I IFN-associated genes at DPI2.

Using the Enrichr server and BioPlanet database, we explored only upregulated genes in the infected neonate compared with the baseline at DPI −7. The radar chart shows a high activation of IFN signaling pathways (–log10(*p*-value)>5) at DPI 2 (peak viral RNA load in oropharyngeal fluids) that remains significantly high at DPI 6. As time progresses, we observe a shift in the key biological process and molecular functions that show activation of transcription, RNA processing, regulation, and degradation (**Figure 4A**). We then focused on DPI 2, which shows a higher IFN pathway activation. Comparing DPI 2 with DPI −7, we found 2,110 differentially expressed genes (DEGs; fold change >2), including 907 that were upregulated and 1,203 downregulated (**Figure 4B**). To follow expression level dynamics of type-I IFN genes throughout the collected blood samples, we then generated a heatmap plotting Log2FC expression values at every time point (**Figure 4C**). Type-I IFN gene expression levels peak at DPI 2 and were maintained at DPI 6 for most of the genes except for IFI35, IFI3, IFI2, SAMHD1, TYK2, and IRF3 (**Figure 4C**). MX1 and IFIT1 were maintained at DPI 12 and 35, unlike the other genes, which decreased at DPI 12, except for IFI35 and IRF3. Finally, we identified confident interactions (at least 4 differently colored lines) between STAT2, TYK2, IRF3, MX1, ISG15, IFIT1, IFIT2, IFIT3, and IFI35 (**Figure 4D**), where STAT2 seems to be the key signal transducer and the transcription activator of some IFN-associated genes (48).

**Figure 5:**
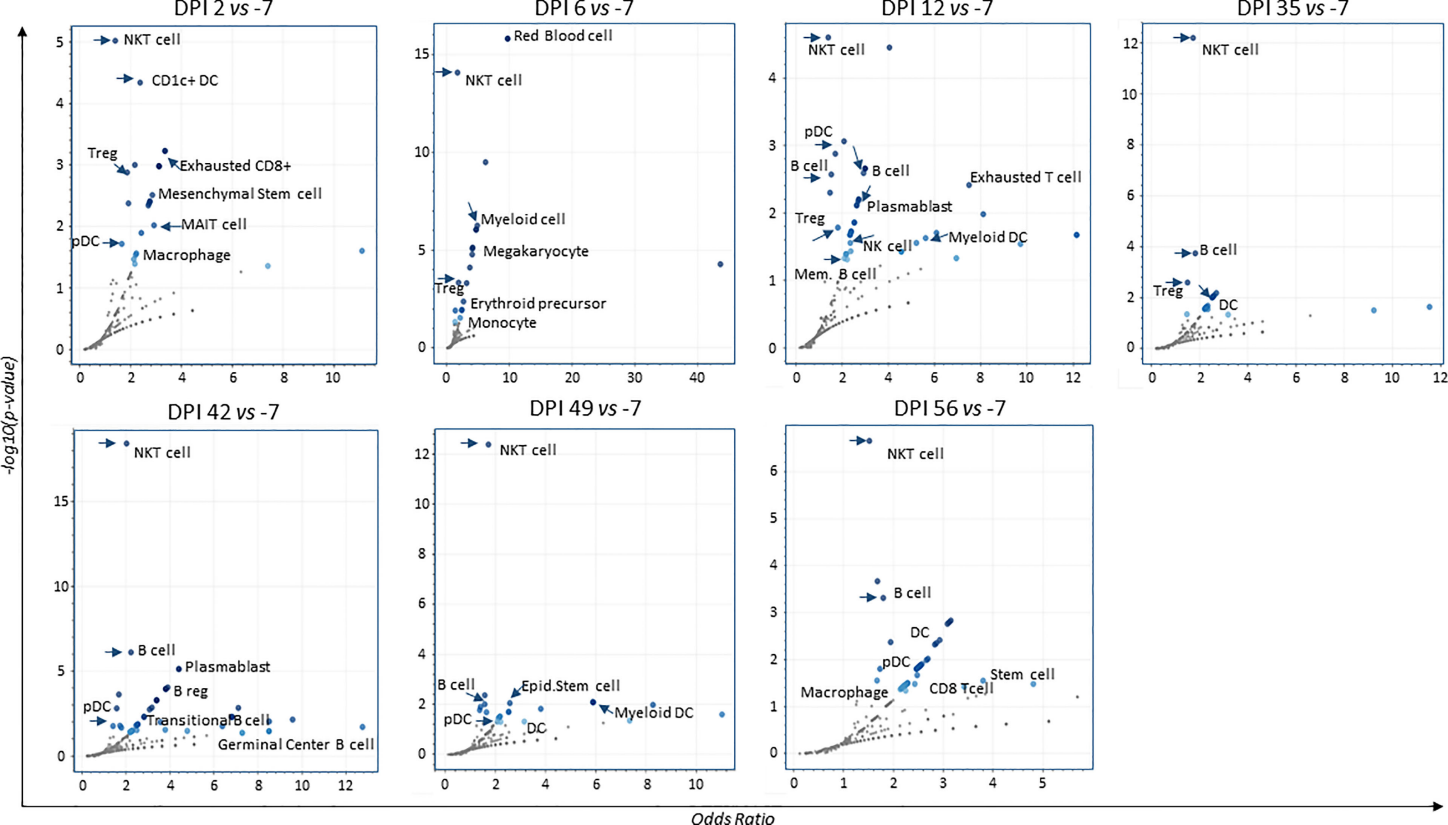
Volcano plots for augmented cell-populations following infection. RNA-sequencing of whole blood cell-populations at each time-point compared to baseline (DPI − 7) (analyses using Enrichr/CellMarkerAugmented2021 and Appyter applications). Each volcano plot shows the significance of each gene-set (dark blue dots). x-Axis measures the odds ratio (0, inf) calculated for the gene-set, while the y-axis gives the –log10(*p*-value) of the gene set. Larger blue points represent significant terms (*p*-value < 0.05); smaller gray points represent non-significant terms. The darker the blue color of a point, the more significant it is.

To assess the presence of gene sets related to cell markers that identify cell populations and determine the quality of the immune response induced after infection and throughout all time points, we used the Enrichr/CellMarkerAugmented2021 and Appyter applications. We generated volcano plots showing the presence and significance of each gene set (dark blue dots, **Figure 5**). As shown in the figure, the presence of natural killer T-cell (NKT) gene sets persisted at each time point (DPI 2, 6, 12, 35, 42, 49, and 56). We also revealed the presence of gene sets related to populations of dendritic cells (DC), regulatory T cells (Treg), and subsets of plasmablast and memory B cells.

Overall, these findings show the development of an early innate response in neonate NHP coinciding with increased viral RNA load at DPI 2.

### SARS-CoV-2-Exposed Neonate Shows a High Humoral B-Cell Response in Whole Blood

We performed flow cytometry analyses of longitudinal whole blood samples to study the dynamics of cell populations before and after SARS-CoV-2 exposure of the neonate. Data from two infected adults were used for comparison. To avoid intrinsic differences between neonate and adult cell count (**Supplementary Figure S2B**), we chose to present the results as a fold increase from the baseline at DPI −7.

**Figure 6:**
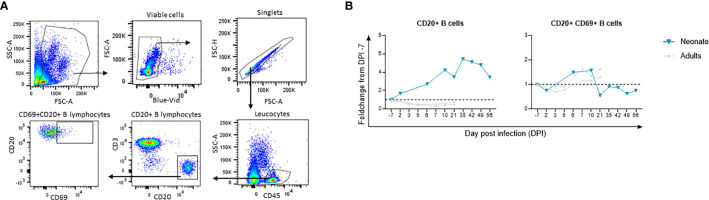
Flow cytometry analyses for longitudinal B cell immune response following infection. Fold-changes from the baseline are shown for each cell population. The neonate is shown in blue line whereas adults (n=2) are shown in grey lines. **(A)** Gating strategy for B cell subsets. **(B)** CD45+CD20+B cells and activated CD69^+^CD20^+^B cell fold change from the baseline. The dashed line represents the baseline (Fold change 1).

White blood cell (WBC) counts results showed no major changes throughout the follow-up in the neonate (**Supplementary Figure S2A**). By flow cytometry, however, we observed a high fold increase of CD45^+^CD20^+^ B cells from the baseline in the neonate compared with adults, which started from DPI 6 and peaked at DPI 35 to then plateau at DPI 42 and 49 before decreasing (**Figure 6**). This increase coincided with B-cell activation (CD69^+^CD20^+^ cells peak at DPI 6 and 10; **Figure 6**) and an increase of SARS-CoV-2 RBD binding IgG and neutralizing antibodies (starting at DPI 35, **Figure 2C**). This increase also coincided with enrichment in B-cell populations starting from DPI 12 and onwards, as shown by RNA-sequencing data (**Figure 5**). Our data are in line with previous studies showing the presence of specific plasmablast B cells as well as neutralizing antibodies in children with low viral RNA load 1 week after disease onset (49). Our results show an increase in humoral B-cell responses in the blood of SARS-CoV-2-exposed neonates.

### SARS-CoV-2-Exposed Neonate Shows Longitudinal Cytokine Changes in Whole Blood, Oropharyngeal, and Rectal Mucosae

Local and systemic innate and adaptive responses were monitored by measuring cytokine concentration in oropharyngeal and rectal fluids, as well as in peripheral blood from the infected neonate at all time points (**Supplementary Table S2**).

**Figure 7:**
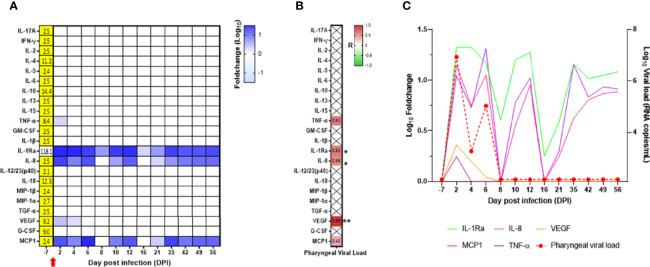
Cytokine analyses in the neonate oropharyngeal compartment. **(A)** Heatmap for the evolution of cytokines over time (in the log of fold change from the baseline). Variations are shown on a blue scale. Basal concentrations (pg/ml) are shown on the left side of the heatmap. Highlighted yellow values correspond to the detection threshold. **(B)** Spearman correlation analysis between SARS-CoV-2 viral RNA load in the oropharyngeal compartment and the kinetics of cytokines in the same compartment was performed. The obtained correlation factors, which have been determined on the whole kinetics, are shown on the heatmap with a red to green scale. ^*^p < 0.04 and ^**^p < 0.003. **(C)** Graph overlapping the kinetics of the cytokines in the log of fold change (left scale) with the oropharyngeal viral RNA load on the logarithmic scale (right scale).

In the oropharyngeal compartment, IL-1Ra, IL-8, VEGF, MCP-1, and TNF-α were increased at DPI 2. As for flow cytometry, the results are presented as a fold increase from the baseline at DPI −7. VEGF and TNF-α levels were relatively weak and transient, whereas IL-1Ra, IL-8, and MCP-1 levels were high and were maintained at different time points following infection (**Figure 7A**; **Supplementary Table S2**). IL-1Ra, IL-8, and VEGF were positively and significantly correlated with the oropharyngeal viral RNA load (**Figure 7B**), as shown by the overlay of synchronized curve kinetics before and after infection (**Figure 7C**).

**Figure 8:**
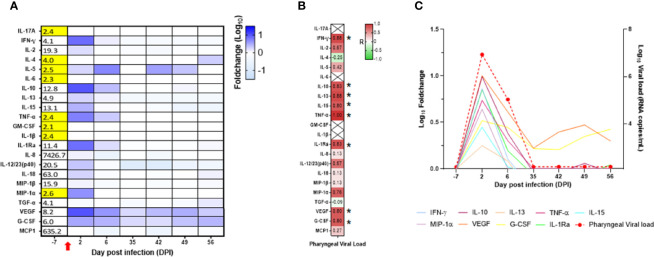
Cytokine analyses in the neonate blood. **(A)** Heatmap representing the evolution of cytokines over time (in the log of fold change from the baseline). Variations are shown on a blue scale. Basal concentrations (pg/ml) are shown on the left side of the heatmap. Highlighted yellow values correspond to the detection threshold. **(B)** Spearman correlation analysis between SARS-CoV-2 viral RNA load in the oropharyngeal compartment neonate and the kinetics of cytokines in the blood. Correlation factors determined on the whole kinetics are shown on the heatmap with a red to green scale. ^*^p < 0.03. **(C)** Graphs overlapping the kinetics of the blood cytokines in the log of fold change (left scale) with the oropharyngeal viral RNA load on the logarithmic scale (right scale).

In the blood, we observed increased levels of IFN-γ, IL-2, IL-5, IL-10, IL-13, IL-15, TNF-α, IL-1Ra, IL-8, IL-12/23, IL-18, MIP-1α, VEGF, and G-CSF with a peak at DPI 2, whereas IL-5 peaked at DPI 6, VEGF and G-CSF remained elevated during the whole follow-up (**Figure 8A**; **Supplementary Table S2**). Compared with the adult control group, the main differences we observed in the neonate were high levels of MCP-1 and IL-8 at baseline and high levels of IL-10 at DPI 2 in the neonate but not in the adults, with only a slight increase at DPI 13 in one animal (**Supplementary Figure S6**).

Unlike the mucosae sites, no blood samples were available at DPI 10, 12, 16, and 21 for cytokine analyses. IL-10, IL-13, IL-15, TNF-α, IFN-γ, IL-1Ra, VEGF, and G-CSF were positively and significantly correlated with the oropharyngeal viral RNA load (**Figure 8B**). As shown in **Figure 8C**, the peak of cytokine levels in blood matched with the peak of oropharyngeal viral RNA load.

**Figure 9:**
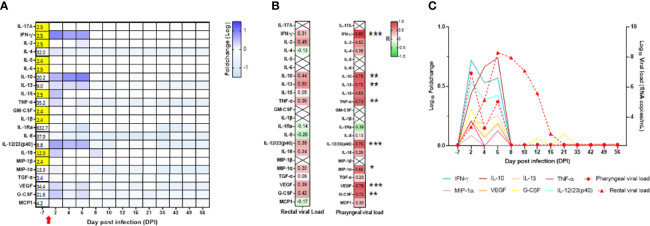
Cytokine analyses in the neonate rectal compartment. **(A)** Heatmap representing the evolution of cytokines over time (in the log of fold-change from the baseline). Variations are shown on a blue scale. Basal concentrations are shown on the left side of the heatmap. Highlighted yellow values correspond to the detection threshold. **(B)** Spearman correlation analysis between SARS-CoV-2 viral RNA load in the rectal (left) or oropharyngeal (right) compartment and the kinetics of cytokines in the rectal compartment. Correlation factors determined on the whole kinetics are shown on the heatmap with a red to green scale. ^*^p < 0.03*; ^**^p < 0.008; ^***^p = 0.0006. **(C)** Graphs overlapping the kinetics of the rectal cytokines in the log of fold change (left scale) with the oropharyngeal and rectal viral RNA load on the logarithmic scale (right scale).

 In the rectal mucosa, levels of IFN-γ, IL-10, and IL-12/IL-23 increased at DPI 2 and were maintained at DPI 4 and DPI 6, whereas IL-4, IL-13, IL-15, TNF-α, IL-18, VEGF, and G-CSF had intermediate levels and returned to baseline level after DPI 6 (**Figure 9A**; **Supplementary Table S2**). In contrast to blood and oropharyngeal cytokines, there was no correlation between rectal cytokines and rectal viral RNA load (**Figure 9B**). However, we observed a positive and significant correlation in rectal cytokines with oropharyngeal viral RNA load (**Figure 9B**). The peak of cytokine levels in rectal mucosa matched with the peak of oropharyngeal viral RNA load (**Figure 9C**). Overall, our results show the presence of an early cytokine increase in the blood, oropharyngeal, and rectal mucosae compartments following SARS-CoV-2 infection.

### Microbiota Composition Changes in Oropharyngeal and Rectal Mucosae Following SARS-CoV-2 Infection

**Figure 10:**
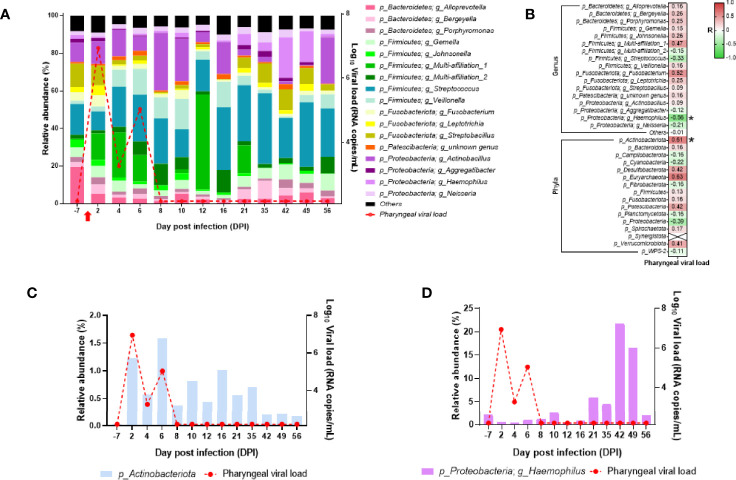
Analyses of the neonate oropharyngeal microbiota composition. **(A)** Bar plots represent the oropharyngeal microbiota composition. Relative abundance is represented for each time point (left scale). The red curve represents the oropharyngeal viral RNA load (logarithmic scale) of SARS-CoV-2 detected in the neonate over time (right scale). p-Firmicutes and g_Multi-affiliation_1 correspond to c_Bacilli and p-Firmicutes; g_Multi-affiliation_2 corresponds to o_Lactobacillales. **(B)** Spearman correlation analysis between the SARS-CoV-2 oropharyngeal viral RNA load and the oropharyngeal microbiota composition. On the heatmap, the correlation factors of each phylum or genus are represented according to the oropharyngeal viral RNA load. Correlation factors are indicated on a red to green scale. ^*^p < 0.036. **(C)** *Actinobacteria* (phylum) relative abundance (left scale) overlapped with oropharyngeal viral RNA load on the logarithmic scale (right scale). **(D)** *Haemophilus* (genus) relative abundance (left scale) overlapped with oropharyngeal viral RNA load on the logarithmic scale (right scale).

We assessed microbiota composition in the oropharyngeal and rectal compartments of the neonate using the V3–V4 16S sequencing. In the oropharyngeal microbiota, the three dominant phyla found were *Firmicutes*, *Proteobacteria*, and *Bacteroides*. Seventeen genera were present at more than 1% of relative abundance throughout the follow-up (**Figure 10A**). The oropharyngeal microbiota composition was similar throughout the follow-up, except at DPI 12 where *Streptococcus* and *Veillonella* genus decreased and *Bacilli* class increased. However, those changes seemed to be independent of the SARS-CoV-2 infection (**Figure 10A**). During the follow-up, some fluctuations were observed in the phylum *Actinobacteriota* and the genus *Haemophilus* that correlated positively and negatively, respectively, with the oropharyngeal viral RNA load (**Figures 10B–D**).

**Figure 11:**
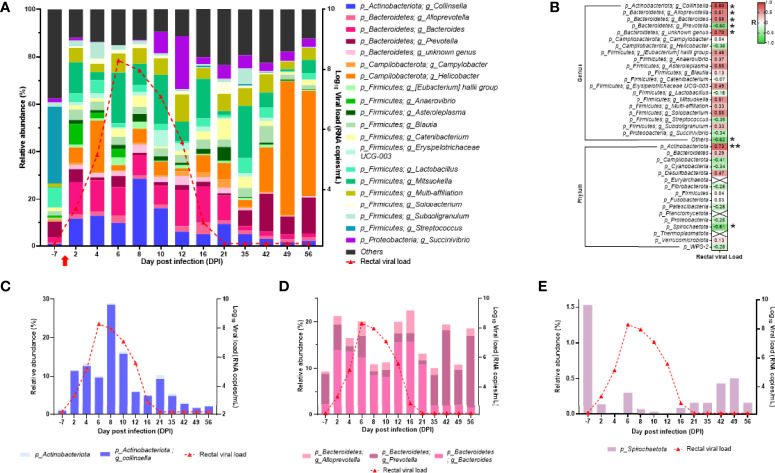
Analyses of the neonate rectal microbiota composition. **(A)** Bar plots represent the rectal microbiota composition. Relative abundance is represented for each time point (left scale). The red curve represents the rectal viral RNA load (logarithmic scale) of SARS-CoV-2 detected in neonate over time (right scale). **(B)** Spearman correlation analysis between the SARS-CoV-2 rectal viral RNA load and the rectal microbiota composition. On the heatmap, the correlation factors of each phylum or genus are represented according to the rectal viral RNA load. Correlation factors are indicated on a red to green scale. ^*^p < 0.03; ^**^p < 0.001. **(C)** *Actinobacteria* (phylum) and *Collinsella* (genus) relative abundance (left scale) overlapped with rectal viral RNA load on the logarithmic scale (right scale). **(D)** *Alloprevotella*, *Bacteroides*, and *Prevotella* (genus) relative abundance (left scale) overlapped with rectal viral RNA load on the logarithmic scale (right scale). **(E)** *Spirochaetota* (phylum) relative abundance (left scale) overlapped with rectal viral RNA load on the logarithmic scale (right scale).

In the rectal microbiota, the three dominant phyla varied over time (**Figure 11A**). In fact, at baseline (DPI −7), the rectal microbiota was dominated by *Firmicutes*, *Bacteroidetes*, and *Proteobacteria*, but during infection (detectable viral RNA load), *Firmicutes*, *Actinobacteriota*, and *Bacteroidetes* become the dominant phyla (**Figure 11A**). After infection (back to undetectable viral RNA load), the dominant phyla were *Campilobacterota*, *Firmicutes*, and *Bacteroidetes* (**Figure 11A**). At the genus level, *Collinsella* and *Bacteroides* increased during infection, and *Helicobacter* and *Prevotella* increased after infection (**Figure 11A**). Many genus or phylum variations were significantly correlated with the rectal viral RNA load (**Figure 11B**). Among the significant correlation, the abundance of the *Actinobacteriota* phylum, mainly driven by the *Collinsella* genus, perfectly followed the viral kinetics (**Figures 11B, C****)**. In the *Bacteroidetes* phylum, three genera were significantly correlated with rectal viral RNA load: *Alloprevotella* and *Bacteroides* with a positive correlation and *Prevotella* with a negative correlation (**Figures 11B, D****)**. *Spirochaetota* variation was negatively correlated with rectal viral RNA load variation (**Figures 11B, E****)**. These results show that the microbiota composition of the oropharyngeal and rectal mucosae changed in the SARS-CoV-2-exposed neonate.

## Discussion

Our pilot study shows that SARS-CoV-2 infection of neonates may represent a suitable model to study early viral and immune response dynamics, with the advantage of having a well-defined onset of an asymptomatic infection, which is difficult to obtain in humans.

We show an effective and asymptomatic infection of the neonate NHP. We observed a correlation of the viral RNA load with the development of an early (DPI 2) innate immune response, accompanied with a balanced IL-10/IFN-γ response in peripheral and mucosal sites. Moreover, these parameters correlated with changes in the oropharyngeal and rectal microbiota profile composition. In the mother, viral RNA loads were low and close to the detection limit, despite the very close contact with SARS-CoV-2-exposed neonate, as demonstrated by RT-qPCR and RNA-sequencing data. In infected adults, we observed a mild asymptomatic infection with transient signs of inflammation, more pronounced than the rapidly resolving form observed in the neonate.

There is a debate on the fact that children are more exposed to common cold coronaviruses and have therefore developed cross-reactive antibodies with some ability to protect them against SARS-CoV-2 (13, 50, 51). However, adults are also exposed and thus should have such immunity (50). Recently, a study reported that in adults and upon exposure to SARS-CoV-2, the influence of pre-existing memory responses in combination with potentially slower activation of the memory B-cell response may contribute to a less rapid and effective antibody response. In contrast, children, who have less-experienced humoral immunity to seasonal coronaviruses, may mount a more specific immune response towards antigens from SARS-CoV-2, by inducing more targeted and Fc functional immunity against SARS-CoV-2 antigens in comparison with adults (14). Whether this mechanism applies here in the SARS-Cov-2-exposed neonate NHP or not is an additional question that can be addressed in future studies. Of note, the neonate in our study was born to a healthy mother in our clean and protected facility.

In our study, we observed an early and high fold increase of B cells in the neonate compared with adults, which started from DPI 6, persisted, and peaked at DPI 35 and DPI 42. The physiological increase of B cells from 1 month described in human neonate (16) might partly explain the observed B-cell expansion. Nonetheless, this increase also coincided with infection-related B-cell enrichment and activation, together with the kinetics of the IgG to RBD and neutralizing antibodies, though the latter were not tested throughout. Antibody titers in the neonate NHP were lower compared with those we previously detected in nonhospitalized COVID-19 adult patients at similar time points postinfection (31).

Furthermore, we observed the presence of gene sets related to some cell populations, namely dendritic, B, and T cells, as assessed by RNA sequencing of whole blood. The presence of NKT gene sets was persistent at every time point, as shown by the volcano plots. These cells are important as they are involved in both innate and adaptive immunity during respiratory infections (52) and have the capacity to not only rapidly produce some key cytokines but also interact with B cells for prolonged antibody responses with isotype switch and affinity maturation, and development of long-lasting B-cell memory (53–55). We also observed an increase in some innate and adaptive cytokines, chemokines, and growth factors. More specifically, IFN-γ but not IL-17 was highly expressed as early as DPI 2 in the neonate’s blood, which confirms previous data obtained in SARS-CoV-2-infected children (13). We also found an increase in IL-10, TNF-α, IL-1Ra, MIP-1α, and to a lesser extent, IL-5, IL-13, IL-15, IL-12/23, IL-18, and G-CSF at DPI 2. Interestingly, this increase correlated with the peak of oropharyngeal viral RNA load. Some of these cytokines, i.e., IFN-γ, IL-10, IL-15, IL-12/23, TNF-α, IL-1Rα, or IL-8, concomitantly increased in rectal and oropharyngeal mucosae, respectively. Increased blood, oropharyngeal, and rectal cytokines correlated with oropharyngeal viral RNA load at DPI 2. The detection of both regulatory (i.e., IL-10) and effector (i.e., IFN-γ) cytokine expression as early as DPI 2 indicates the existence of a balanced immune response very early following pathogen exposure. This increase of IL-10 was not observed in the blood of adult animals, which is consistent with the tolerogenic profile of the neonate immune system that might participate in the control of potential excessive responses.

Importantly, by using an RNA-sequencing-based approach, we were able to show the presence of an early innate immune response in the infected neonate, but not the mother. In particular, we found a strong IFN gene signature as early as DPI 2 compared with the baseline. Type-I interferon signatures included MX1, IFI2, IFI3, IFI27, IFI44, and ISG15 key genes. Genes’ expression was elevated and persistent over DPI 6 and even DPI 12 for some of them. These data are in line with previous studies from SARS-CoV-2-infected children (13, 14) and https://doi.org/10.1101/2022.02.12.480218, and interestingly enough, also in line with other pediatric infections such as respiratory syncytial and influenza viruses (56).

Although the exact mechanism leading to better and faster disease resolution in children remains unclear, it is tempting to suggest that the robust innate response that develops very early following SARS-CoV-2 infection, as shown in this study and previous reports (10, 13, 14, 57), plays a major role in rapid disease resolution. Moreover, the presence of persistent NKT cells very early following infection, with their maintenance at every time point, suggests their crucial role in the early production of cytokines such as IFN-γ. This early innate response probably led to disease resolution with no need for mounting a high cellular response, as demonstrated by our results on T-cell populations’ dynamics. These results should be reproduced in a larger cohort of neonate NHP. Future studies are underway to assess the potential role of NKT cells in early events of SARS-CoV-2 infection in humans/children.

Given that microbiota plays an important role in shaping the immune responses (58) and that many factors may be responsible for variation in microbiota composition, such as food or exposure to pathogens (59), we sought to assess microbiota composition before and after SARS-CoV-2-neonate NHP exposure, and determine whether we could identify virus-specific bacteria profiles. In adult macaques and humans, SARS-CoV-2 infection causes a transient variation in gut microbiota composition and inflammatory profile (60, 61).

Our results showed some variations of the oropharyngeal microbiota composition, with an increase of the *Actinobacteriota* that correlated with the viral RNA load and of *Haemophylus* spp. from DPI 21 to DPI 49. This increase of *Haemophylus* spp. was reported in children 3 months after antibiotics treatment (62), suggesting that it could be an indicator of dysbiosis in the oropharyngeal microbiota of the neonate. The SARS-CoV-2 effects on the rectal microbiota composition were more pronounced, and the number of the bacterial genus was directly impacted by SARS-CoV-2 infection. While most genera return to normal after infection, *Helicobacter* spp. stayed high. Usually, this genus is not detected in the rectal microbiota of macaques (63), while in humans, it was correlated with several intestinal and hepatobiliary diseases (64). We can therefore reason that SARS-CoV-2 infection in the neonate macaque caused dysbiosis in oropharyngeal and rectal mucosae. The consequences of such dysbiosis on the implementation of immunity and long-term immunity effects should be investigated.

Our study has a few limitations. Only one neonate NHP was studied, which was mainly due to the urgent need for rapidly developing a pediatric SARS-CoV-2 neonate model when the COVID-19 pandemic started (65–67). In a context of strong pressure for SARS-CoV-2 studies on macaque supply, only one animal was available. Consequently, a noninfected SARS-CoV-2 neonate NHP was also unavailable to compare the immune responses and the evolution of the microbiota. Another limitation is the lack of bronchoalveolar lavage and larger blood samples from the neonate for further virological and extended immunological explorations due to ethical reasons. Indeed, larger cohorts are needed to confirm the results from this pilot study.

In summary, we have identified an early innate response in a SARS-CoV-2-infected neonate NHP with mild disease. This response included the presence of an antiviral type-I IFN genes signature, a persistent and lasting NKT cell population, a balanced peripheral and mucosal IFN-γ/IL-10 cytokine response, and a high increase in B cells that was accompanied with anti-SARS-CoV-2 IgG response. These results suggest an age-dependent differential immune response to SARS-CoV-2 infection will have to be confirmed in a larger number of animals to explore the pathogenesis in children.

## Data Availability Statement

The datasets presented in this study can be found in online repositories. The names of the repository/repositories and accession number(s) can be found below: NCIO BioProject, PRJNA800739.

## Ethics Statement

The animal study was reviewed and approved by CEA and complies with the French national regulation (facility authorization number #D92-032-02), the European Directive 2010/63/EU, and the Standards for Human Care and Use of Laboratory Animals (OLAW animal welfare assurance number #A5826-01, United States). The study was approved by the local ethics committee (CEtEA#44) and the French Research, Innovation, and Education Ministry under registration number APAFIS #24434-20200030216532863 V3.

## Author Contributions

C-MF, MG, CP, NN, RG, EM, and NS conceived, designed, performed experiments, analyzed data, supervised and managed the project, provided funding, and wrote the manuscript (original draft). FR, MC, RF, TN, PM, IM, MT, QS, ND, and JW performed experiments and analyzed the results. VL and GS provided funding and assisted in data interpretation. ND-B supervised the RT-PCR and cytokine experiments. A-SG supervised the flow cytometry experiments. All authors contributed to the reviewing and editing of the final manuscript.

## Funding

This work was supported by the “Programme Investissements d’Avenir” (PIA) managed by the ANR under reference ANR-11-INBS-0008, funding the Infectious Disease Models and Innovative Therapies (IDMIT, Fontenay-aux-Roses, France) infrastructure, and ANR-10-EQPX-02-01, funding the FlowCyTech facility (IDMIT, Fontenay-aux-Roses, France). The Fondation Bettencourt Schueller and the Region Ile-de-France contributed to the implementation of IDMIT’s facilities and imaging technologies. The NHP model of SARS-CoV-2 infection have been developed thanks to the support from REACTing, the Fondation pour la Recherche Medicale (FRM; AM-CoV-Path), and the European Infrastructure TRANSVAC2 (730964). The virus stock used in NHPs was obtained even through the EVAg platform (https://www.european-virus-archive.com/), funded by H2020 (653316). The work performed at IRCCS Ospedale San Raffaele (OSR) was funded by Program Project COVID-19 OSR-UniSR and Ministero della Salute (COVID-2020-12371617). The funders had no role in the design of the study, data collection or interpretation, or the decision to submit the work for publication. We thank Foundation Dormeur, Vaduz for the donation of laboratory instruments relevant to this project to the Viral Evolution and Transmission Unit at OSR.

## Conflict of Interest

Author Natalia Nunez was employed by Life and Soft, Fontenay-aux-Roses, France.

The remaining authors declare that the research was conducted in the absence of any commercial or financial relationships that could be construed as a potential conflict of interest.

## Publisher’s Note

All claims expressed in this article are solely those of the authors and do not necessarily represent those of their affiliated organizations, or those of the publisher, the editors and the reviewers. Any product that may be evaluated in this article, or claim that may be made by its manufacturer, is not guaranteed or endorsed by the publisher.

## Supplementary Material

Supplementary Figure 1 Clinical parameters of the neonate/mother pair. **(A)** Temperature, oxygen saturation, respiratory and heart rate for the neonate (blue line) and its mother (orange line). The horizontal dashed line(s) is for the physiological accepted values. **(B)** Neonate body weight. **(C)** Biochemical parameters values for ASAT/ALAT, creatinine, CRP, haptoglobin, LDH, total protein, troponin I and urea. The vertical red dashed line indicates viral inoculation at DPI 0.

Supplementary Figure 2 Hematological parameters of the neonate/mother pair. **(A)** Hemoglobin concentration and absolute numbers of white blood cells, neutrophils, lymphocytes, red blood cells, monocytes, eosinophils, basophils in the neonate (blue line) and its mother (orange line). The vertical red dashed line indicates viral inoculation at DPI 0. **(B)** Evolution of the leukocytes subpopulations count in the neonate blood during the first ten weeks of life compared to adult profile.

Supplementary Figure 3 Clinical parameters of infected adults. **(A)** Temperature, oxygen saturation, respiratory and heart rate of seven adults from three studies (pink, green and blue lines). The horizontal dashed line(s) is for the physiological accepted values. **(B)** Biochemical parameters values for ASAT/ALAT, creatinine, CRP, haptoglobin, LDH, total protein and troponin I. The horizontal dashed lines stand for median with standard deviations according to reference values (68, 69). The vertical red dashed line indicates viral inoculation at DPI 0.

Supplementary Figure 4 Hematological parameters of infected adults. Hemoglobin concentration and absolute numbers of white blood cells, neutrophils, lymphocytes, red blood cells, monocytes, eosinophils, basophils in adults from three studies (pink, green and blue lines). The horizontal dashed lines stand for median with standard deviations according to reference values (68, 69). The vertical red dashed line indicates viral inoculation at DPI 0.

Supplementary Figure 5 SARS-CoV-2 infection in adults. Viral RNA load measured by RT-qPCR in the fluidic compartments. The limit of detection was estimated at 2.37 log10 copies per ml and the limit of quantification was estimated at 3.37 log10 copies per ml (dotted horizontal line). Macaques were included in three studies with specific SARS-CoV-2 inoculated doses: MM1-4 received 1x10^5^ CFU (pink), MM5 received 1x10^6^ CFU (green) and MM6-7 received 1x10^7^ CFU (blue).

Supplementary Figure 6 Cytokine analyses in blood from infected adults. Heatmap representing the evolution of cytokines over-time in three adults (in log of fold-change from the baseline). Variations are shown on a blue scale. Basal concentrations (pg/ml) are shown on the left side of the heatmap. Highlighted yellow values correspond to the detection threshold.

Supplementary Table 1 Individual data of infected adult rhesus macaques. SARS-CoV-2 inoculation protocol and sampling planning for mucosal swabs and blood collection of seven adult females. MM1-4 received 1x10^5^ CFU and had a 28 DPI follow-up (pink), MM5 received 1x10^6^ CFU and had a 4 DPI follow-up (green), and MM6-7 received 1x10^7^ CFU with 28 DPI follow-up (blue). Samples included heparin-lithium blood and fluids from nasal, rectal and broncho-alveolar lavages compartments.

Supplementary Table 2 Cytokine concentrations in the neonate compartments. Cytokine quantification was performed in blood, oropharyngeal and rectal fluids with the Milliplex MAP kit for NHP, based on Luminex^®^ xMAP multiplex technology.
