# Supplementary material for: A Case Study to Dissect Immunity to SARS-CoV-2 in a Neonate Nonhuman Primate Model
Source: Front Immunol. 2022 May 4;13:855230. doi: 10.3389/fimmu.2022.855230 (PMC9114777; doi:10.3389/fimmu.2022.855230)
Supplement: Supplementary Figure 1 — Clinical parameters of the neonate/mother pair. (A) Temperature, oxygen saturation, respiratory and heart rate for the neonate (blue line) and its mother (orange line). The horizontal dashed line(s) is for the physiological accepted values. (B) Neonate body weight. (C) Biochemical parameters values for ASAT/ALAT, creatinine, CRP, haptoglobin, LDH, total protein, troponin I and urea. The vertical red dashed line indicates viral inoculation at DPI 0. [file Presentation_1.pptx]

## Slide 1
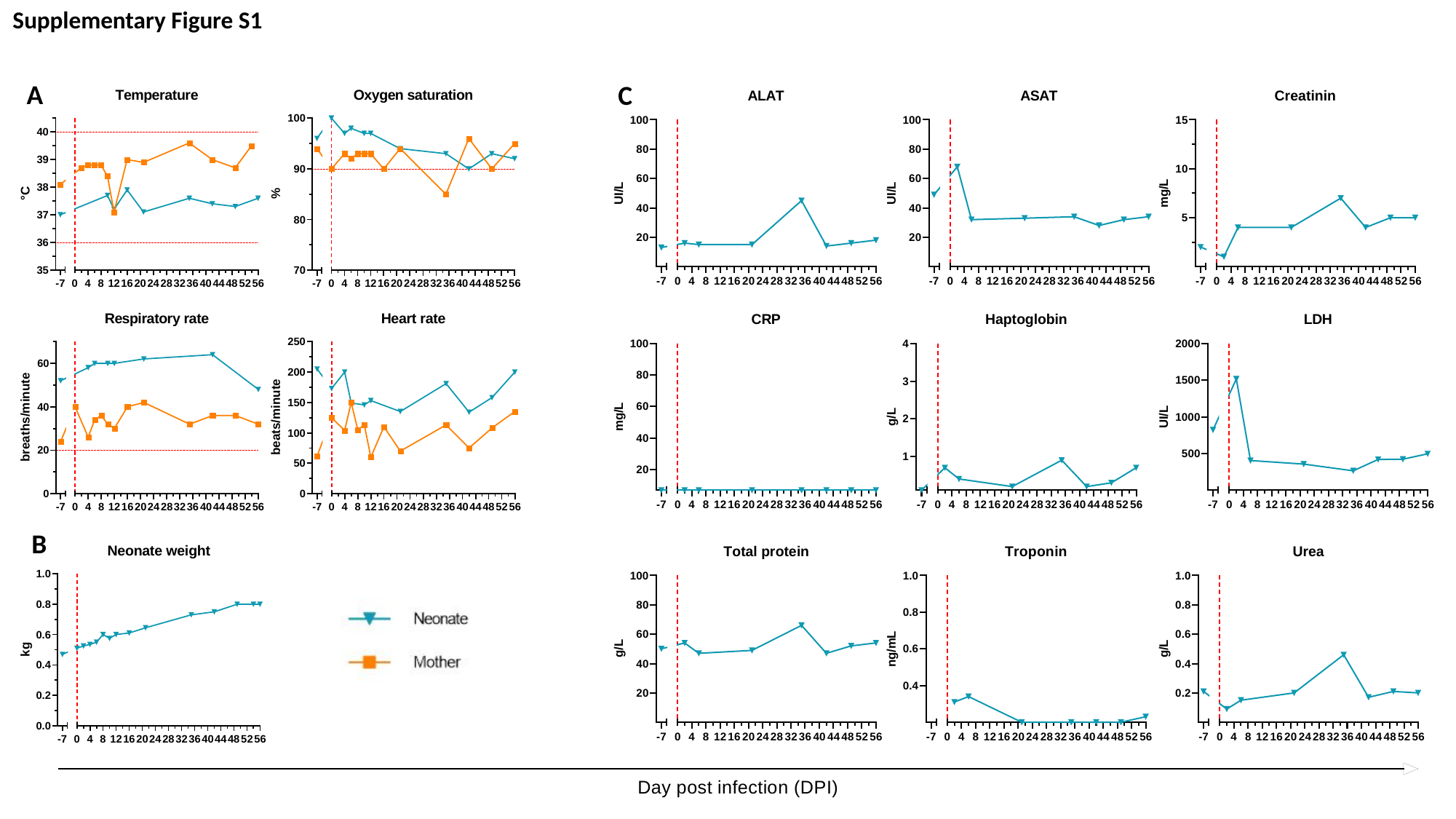

Supplementary Figure S1
A
C
B

## Slide 2
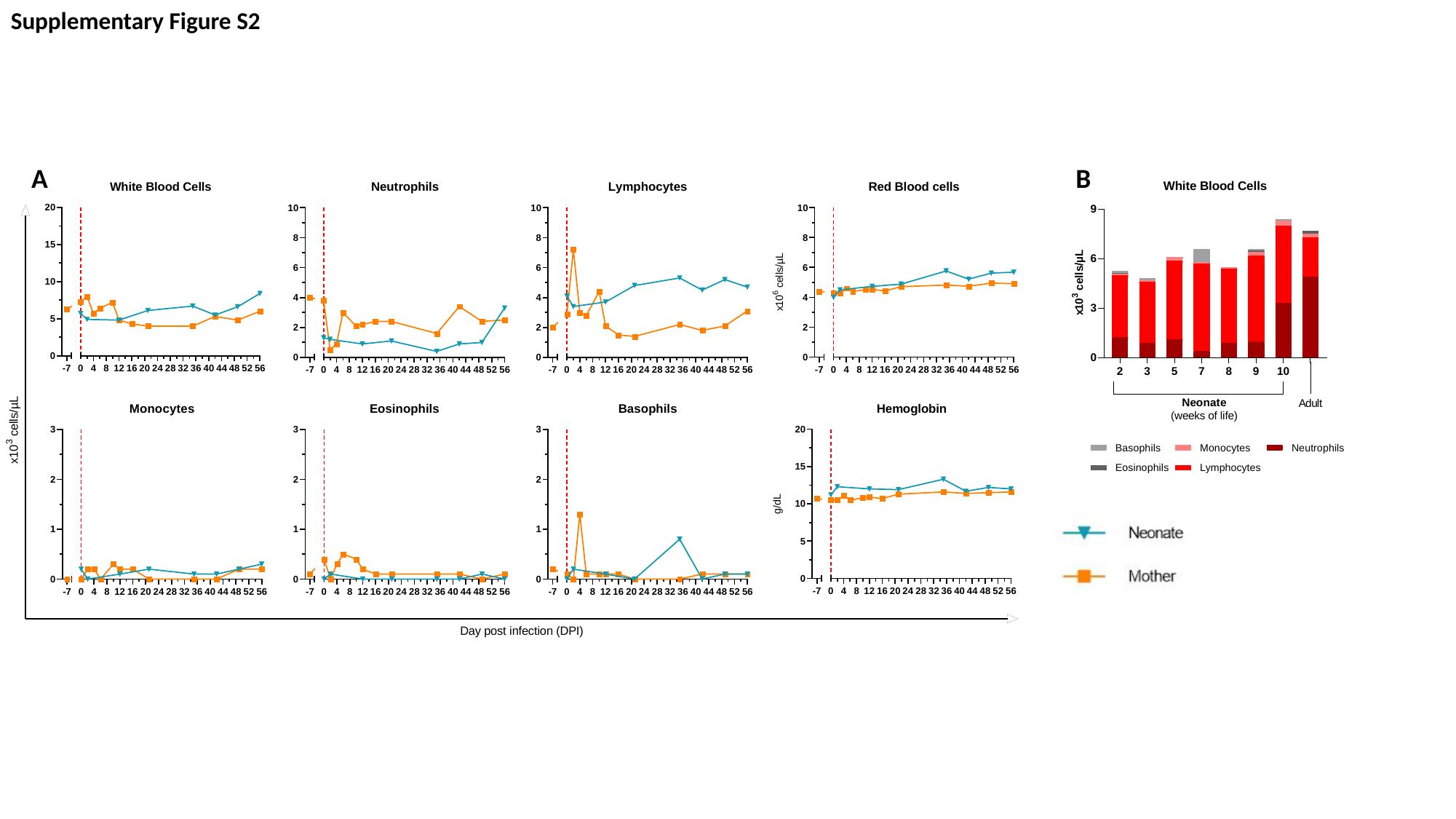

Supplementary Figure S2
A
B

## Slide 3
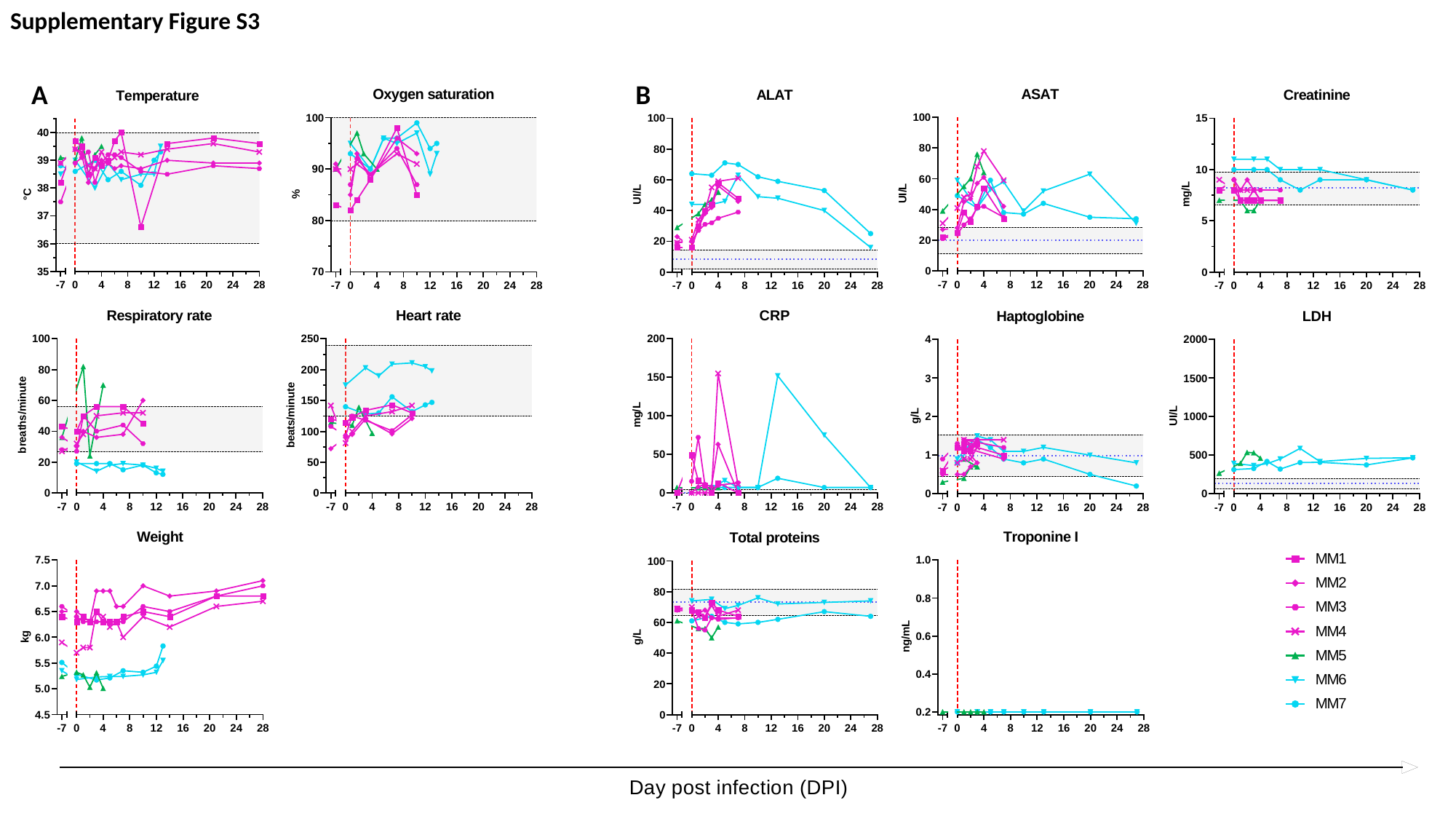

Supplementary Figure S3
A
B

## Slide 4
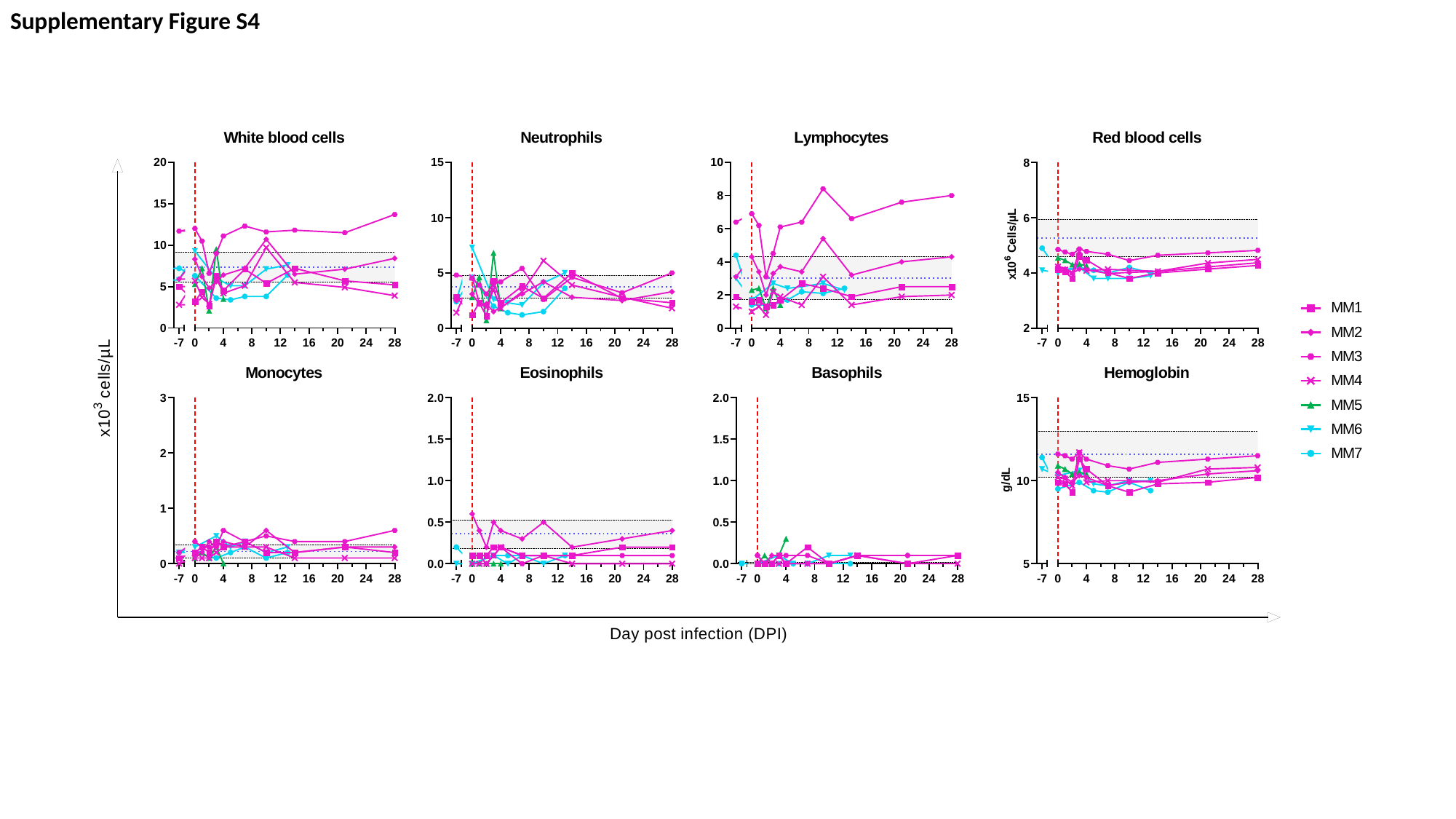

Supplementary Figure S4

## Slide 5
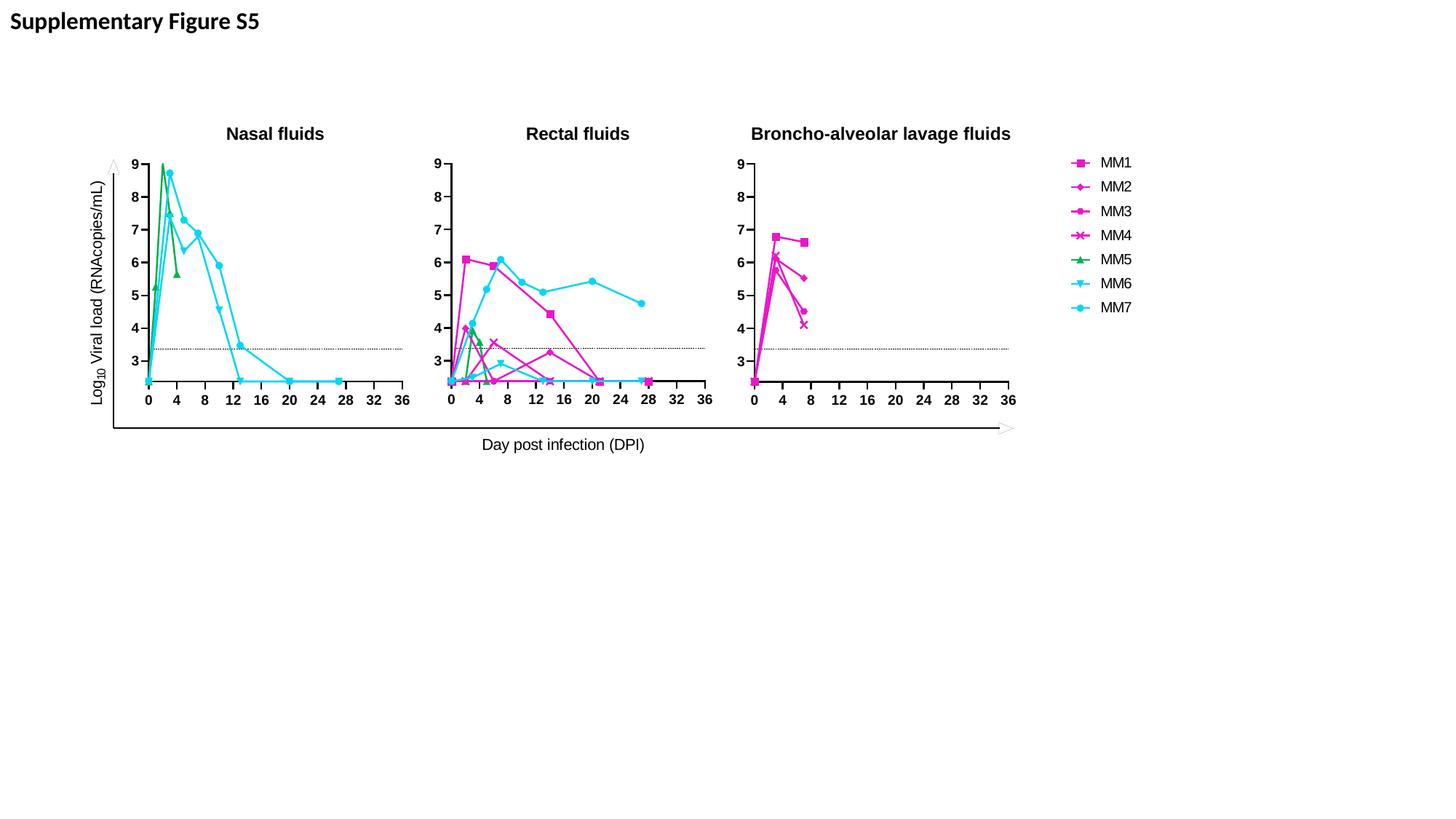

Supplementary Figure S5

## Slide 6
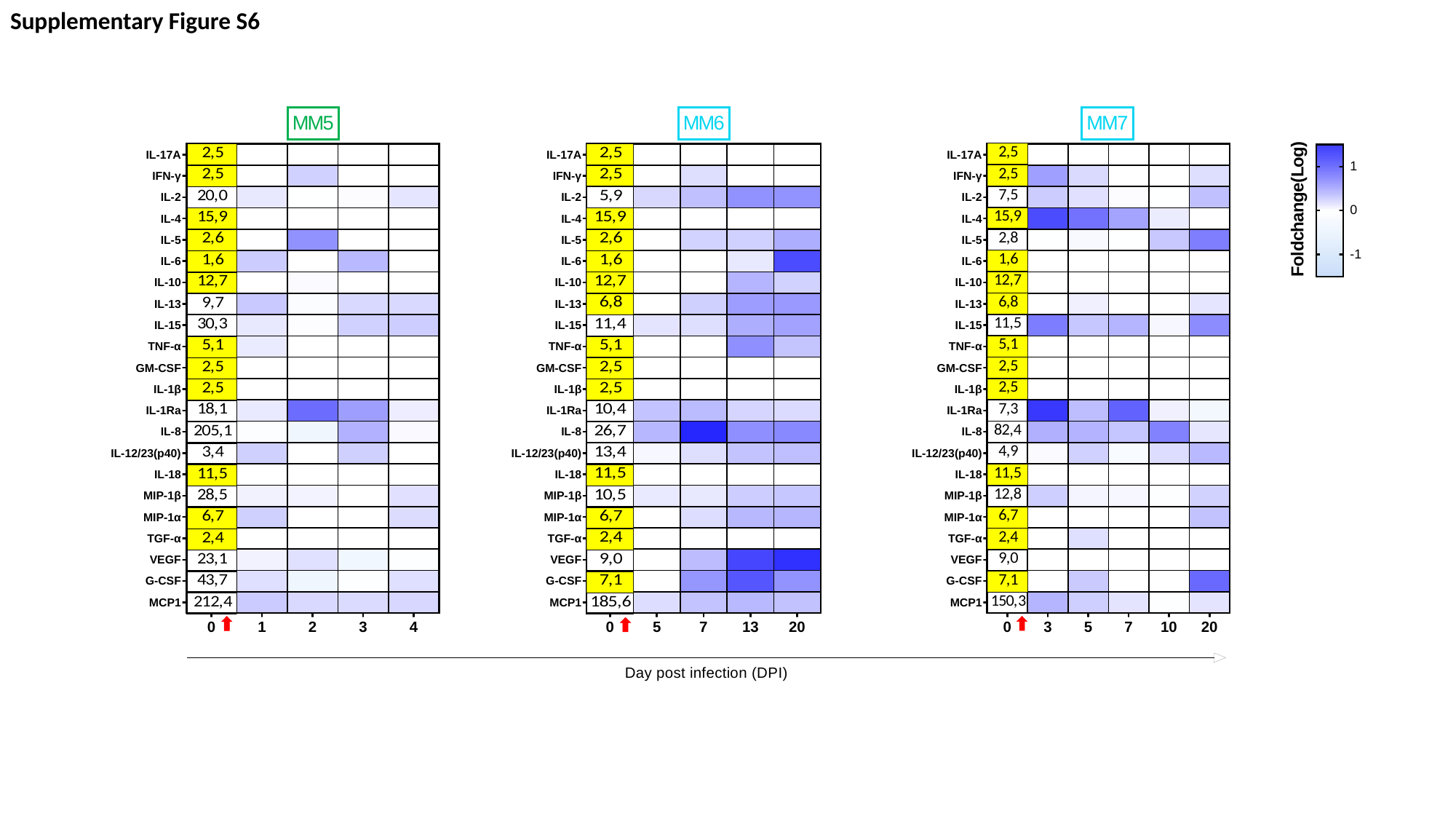

Supplementary Figure S6

## Slide 7
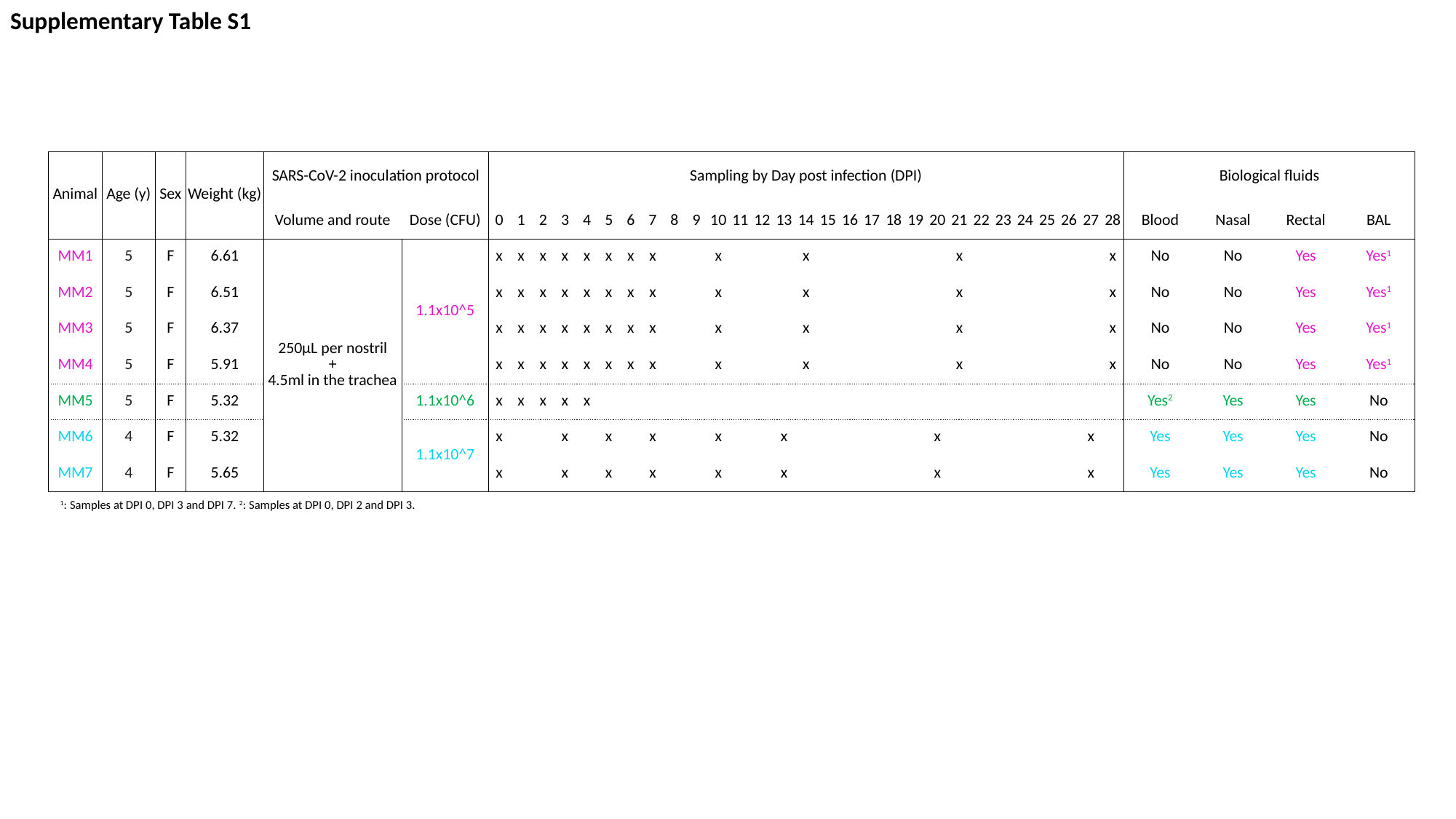

Supplementary Table S1
| Animal | Age (y) | Sex | Weight (kg) | SARS-CoV-2 inoculation protocol | | Sampling by Day post infection (DPI) | | | | | | | | | | | | | | | | | | | | | | | | | | | | | Biological fluids | | | |
| --- | --- | --- | --- | --- | --- | --- | --- | --- | --- | --- | --- | --- | --- | --- | --- | --- | --- | --- | --- | --- | --- | --- | --- | --- | --- | --- | --- | --- | --- | --- | --- | --- | --- | --- | --- | --- | --- | --- |
| | | | | Volume and route | Dose (CFU) | 0 | 1 | 2 | 3 | 4 | 5 | 6 | 7 | 8 | 9 | 10 | 11 | 12 | 13 | 14 | 15 | 16 | 17 | 18 | 19 | 20 | 21 | 22 | 23 | 24 | 25 | 26 | 27 | 28 | Blood | Nasal | Rectal | BAL |
| MM1 | 5 | F | 6.61 | 250µL per nostril + 4.5ml in the trachea | 1.1x10^5 | x | x | x | x | x | x | x | x | | | x | | | | x | | | | | | | x | | | | | | | x | No | No | Yes | Yes1 |
| MM2 | 5 | F | 6.51 | | | x | x | x | x | x | x | x | x | | | x | | | | x | | | | | | | x | | | | | | | x | No | No | Yes | Yes1 |
| MM3 | 5 | F | 6.37 | | | x | x | x | x | x | x | x | x | | | x | | | | x | | | | | | | x | | | | | | | x | No | No | Yes | Yes1 |
| MM4 | 5 | F | 5.91 | | | x | x | x | x | x | x | x | x | | | x | | | | x | | | | | | | x | | | | | | | x | No | No | Yes | Yes1 |
| MM5 | 5 | F | 5.32 | | 1.1x10^6 | x | x | x | x | x | | | | | | | | | | | | | | | | | | | | | | | | | Yes2 | Yes | Yes | No |
| MM6 | 4 | F | 5.32 | | 1.1x10^7 | x | | | x | | x | | x | | | x | | | x | | | | | | | x | | | | | | | x | | Yes | Yes | Yes | No |
| MM7 | 4 | F | 5.65 | | | x | | | x | | x | | x | | | x | | | x | | | | | | | x | | | | | | | x | | Yes | Yes | Yes | No |
1: Samples at DPI 0, DPI 3 and DPI 7. 2: Samples at DPI 0, DPI 2 and DPI 3.

## Slide 8
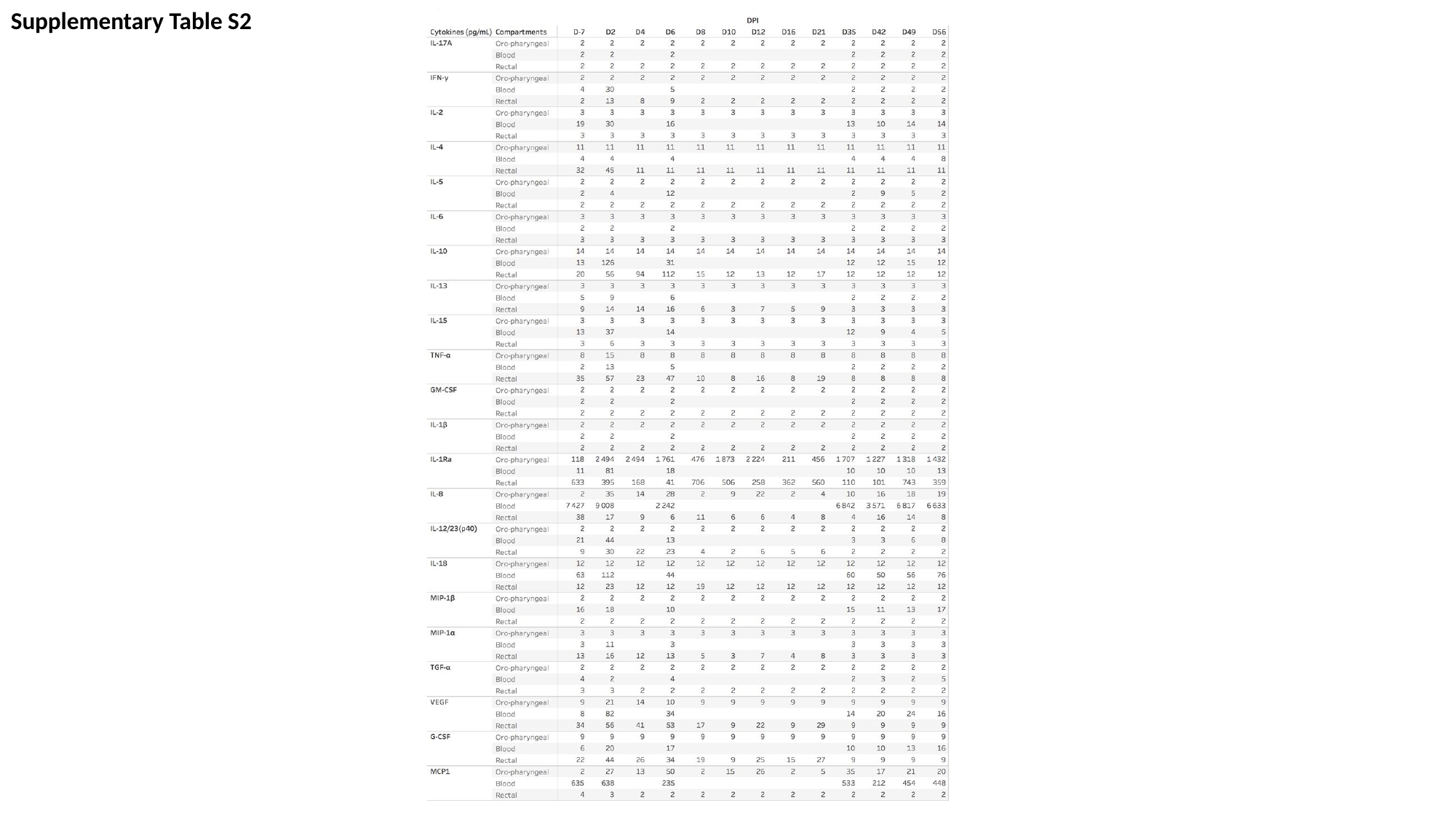

Supplementary Table S2
